# Principles of metabolome conservation in animals

**DOI:** 10.1073/pnas.2302147120

**Published:** 2023-08-21

**Authors:** Orsolya Liska, Gábor Boross, Charles Rocabert, Balázs Szappanos, Roland Tengölics, Balázs Papp

**Affiliations:** ^a^Hungarian Centre of Excellence for Molecular Medicine - Biological Research Centre Metabolic Systems Biology Lab, 6728 Szeged, Hungary; ^b^National Laboratory of Biotechnology, Synthetic and System Biology Unit, Institute of Biochemistry, Biological Research Centre, Eötvös Loránd Research Network, 6726 Szeged, Hungary; ^c^Doctoral School of Biology, University of Szeged, 6726 Szeged, Hungary; ^d^Department of Biology, Stanford University, Stanford, City of Palo Alto, CA 94305-5020; ^e^Inria, 78150 Rocquencourt, 69100 Villeurbanne, France; ^f^Organismal and Evolutionary Biology Research Programme, University of Helsinki, 00014 Helsinki, Finland; ^g^Institute for Computational Cell Biology, Heinrich-Heine Universität, 40225 Düsseldorf, Germany; ^h^Department of Biotechnology, University of Szeged, 6726 Szeged, Hungary; ^i^Metabolomics Lab, Core facilities, Biological Research Centre, Eötvös Loránd Research Network, 6726 Szeged, Hungary; ^j^National Laboratory for Health Security, Biological Research Centre, Eötvös Loránd Research Network, 6726 Szeged, Hungary

**Keywords:** molecular evolution, metabolic networks, systems biology, phylogenetic comparative method, neutral evolution

## Abstract

Metabolite levels shape cellular physiology and disease susceptibility, yet the general principles governing metabolome evolution are largely unknown. Here, we introduce a measure of conservation of individual metabolite levels among related species. By analyzing multispecies tissue metabolome datasets in phylogenetically diverse mammals and fruit flies, we show that conservation varies extensively across metabolites. Three major functional properties, metabolite abundance, essentiality, and association with human diseases predict conservation, highlighting a striking parallel between the evolutionary forces driving metabolome and protein sequence conservation. Metabolic network simulations recapitulated these general patterns and revealed that abundant metabolites are highly conserved due to their strong coupling to key metabolic fluxes in the network. Finally, we show that biomarkers of metabolic diseases can be distinguished from other metabolites simply based on evolutionary conservation, without requiring any prior clinical knowledge. Overall, this study uncovers simple rules that govern metabolic evolution in animals and implies that most tissue metabolome differences between species are permitted, rather than favored by natural selection. More broadly, our work paves the way toward using evolutionary information to identify biomarkers, as well as to detect pathogenic metabolome alterations in individual patients.

## Significance

Metabolites are central players in biochemical reaction networks, and their concentrations shape cellular physiology and disease risks. However, the general principles driving the evolution of metabolite concentrations are essentially unexplored. Here, using cross-species comparisons of metabolomes and biochemical modeling, we report simple rules that universally dictate the evolutionary conservation of metabolite levels in animals. We identified three main factors, metabolite abundance, essentiality, and association with human diseases, that predict evolutionary conservation well. Remarkably, biomarkers of metabolic diseases can be distinguished from other metabolites simply based on evolutionary conservation, without requiring any prior clinical knowledge. This study opens the way to exploit evolutionary information to evaluate the clinical significance of metabolite alterations in humans.

Metabolites are intermediates of biochemical pathways as well as regulators of enzymes and nonenzymatic proteins (1). Accordingly, intracellular metabolite concentrations are key quantities that affect the rates of metabolic reactions (fluxes) and regulate various layers of cellular organization (2, 3). Consequently, metabolite dysregulation underlies various human diseases, from metabolic disorders to cancer (4, 5). Given the tight associations between metabolite levels and cellular physiology, it is often supposed that evolutionary changes in the metabolome contribute to phenotypic differences between species (6, 7). Indeed, shifts in specific metabolite levels have been associated with phenotypic evolution in both mammals (6, 7) and plants (8, 9). However, the general principles driving metabolome evolution remain largely unexplored. Metabolite levels show broad similarities between cells of distantly related organisms (10) and obey simple optimality principles (11, 12), indicating widespread natural selection to preserve them. In fact, metabolome-altering mutations continuously occur during evolution, with harmful ones likely being eliminated by natural selection, affecting patterns of metabolome variation among species. Thus, elucidating the evolutionary forces shaping the metabolome has relevance for a better understanding of the organization of cellular metabolism, and for human health as well.

Here, we propose that the functional role and biochemical properties of metabolites influence the amount of permissible changes in their levels during evolution. Consequently, metabolites that are more strongly constrained by the requirement for proper cellular function, i.e., subject to stronger functional constraints, should be more evolutionarily conserved in their levels. This is analogous to the well-established phenomenon that proteins evolving under stronger functional constraints are more conserved in their sequences (13, 14). Furthermore, just as sequence conservation informs on deleterious genetic variants (15, 16), conservation of metabolite levels should inform on the health impact of metabolite changes.

To test this hypothesis and to gain mechanistic insights into metabolome conservation, we combined phylogenetic analysis of metabolomics data with systems biology modeling. We primarily focused on mammals due to their relevance for human health and the availability of comprehensive multispecies metabolomics data covering a broad phylogenetic range. Specifically, a previous study quantified the relative levels of ~150 metabolites in four major organs of 26 mammalian species, spanning an evolutionary period of ~200 My (7). Our study revealed that the extent of conservation of individual metabolite levels is largely uniform across mammalian lineages, but varies extensively across metabolites. Such variation in conservation results from the differing amounts of functional constraints across metabolites, and is primarily determined by metabolite abundance (i.e., absolute concentration), as evidenced by an independent dataset on absolute metabolite levels in mice (17). Systems modeling revealed that highly abundant metabolites are more closely correlated with key fluxes in the metabolic network, explaining their strong conservation. Remarkably, evolutionary conservation is predictive of the disease-involvements of metabolites, confirming that high conservation implies large fitness impacts in human. Finally, we demonstrated that metabolome evolution is governed by similar rules in the distantly related *Drosophila* genus. Overall, our work offers a universal conceptual framework of metabolome conservation that informs on the disease association of metabolites.

## Results

### Extensive Variation in Evolutionary Conservation of Metabolite Levels.

**Fig. 1.**
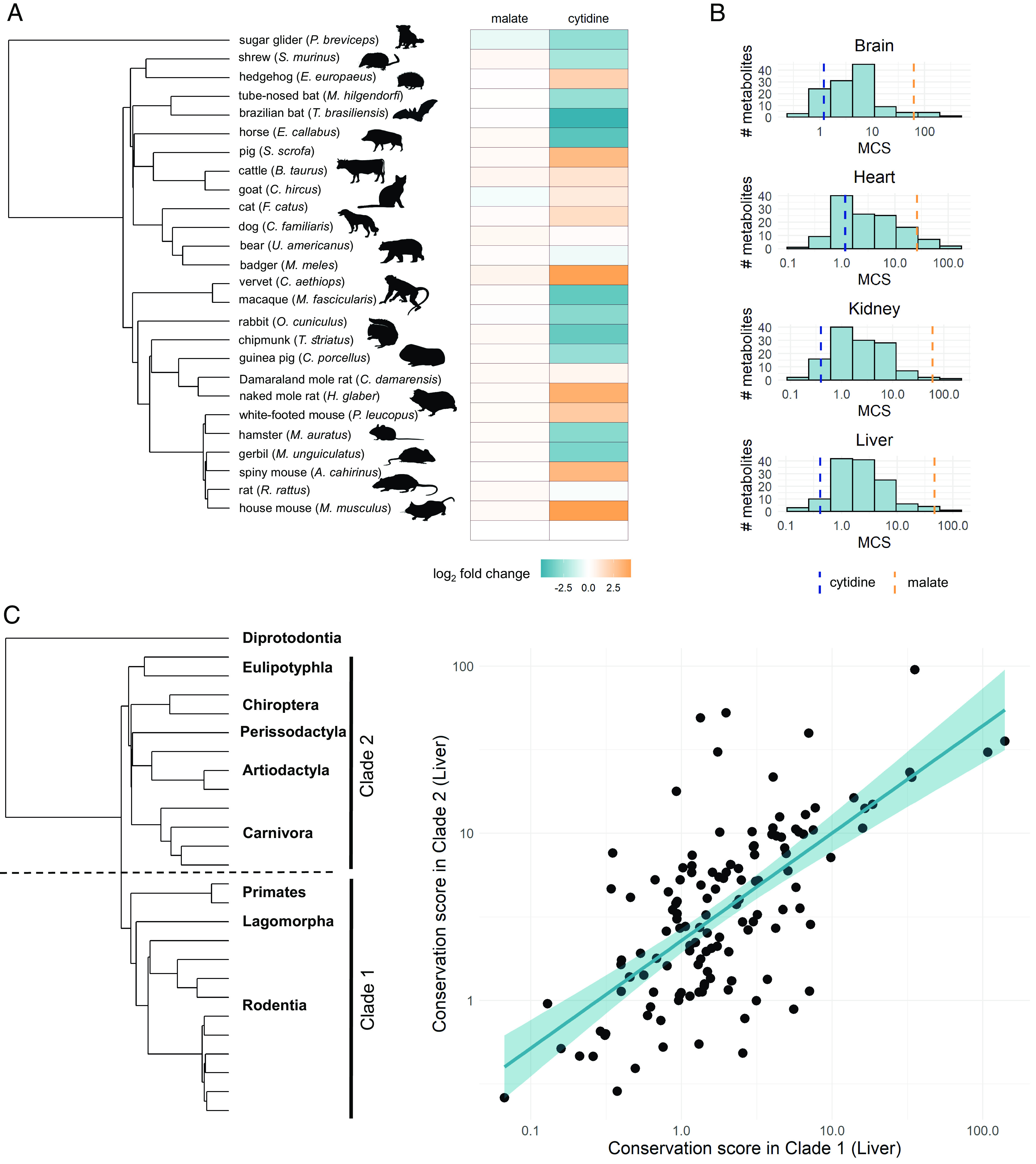
Metabolites differ widely in their conservation score during mammalian evolution. (*A*) Phylogeny of 26 mammals and heatmap illustrating how metabolite levels in liver vary among species (log_2_ fold-change compared to mouse), exemplified by two metabolites: Malate shows highly similar levels in all species, while cytidine is highly variable. (*B*) Distributions of MCSs in each organ, as inferred from all 26 species (logarithmic scale). The vertical dashed lines show the MCSs of the two example metabolites from panel *A* (cytidine–blue, malate–yellow). (*C*) Conservation scores (liver) calculated for two independent clades of the tree show a strong correlation (Pearson’s r = 0.67, *P* = 1.1e−18, N = 132). Line depicts the fitted linear regression. Similar results are obtained for other organs (*SI Appendix*, Fig. S6). The tree on the left depicts the two independent clades of mammals for which conservation scores were inferred, with the names of the constituents’ taxonomical orders.

To systematically investigate the evolutionary conservation of metabolite levels, we first analyzed a published dataset of mammalian metabolomes within a phylogenetic framework. The dataset contains the relative levels of 139 nonlipid metabolites in four organs (brain, kidney, heart, and liver) across 26 mammalian species, representing nine taxonomical orders (7) (Fig. 1*A*). The dataset covers a wide range of central metabolic pathways, including amino acid, carbohydrate, energy, and cofactor metabolism, and represents the most quantitative and phylogenetically diverse multispecies comparison of metabolomes to date. Therefore, it is particularly well-suited to interrogate the general principles of metabolome evolution.

The magnitude of metabolite-level fold-change differences among the 26 species varies widely across metabolites in all studied organs (*SI Appendix*, Fig. S1). For example, in liver, cytidine level varies up to 529-fold among species, while malate shows highly similar levels in all species, with less than 2.3-fold differences (Fig. 1*A*). This pattern suggests extensive variation in the evolutionary conservation of metabolite levels among different metabolites. To more rigorously estimate the degree of evolutionary conservation, while also accounting for species phylogeny, we introduce a score that captures the extent of conservation of metabolite levels along the phylogeny for each metabolite and for each organ (*Methods*, Dataset S1A). This metabolite conservation score (MCS) is based on the widely used Brownian motion (BM) model of evolution, which provides a simple and informative measure of evolutionary rate for quantitative traits (18). We define MCS as the inverse of the rate of evolutionary change in metabolite level (*Methods*). Importantly, MCS requires information only on the relative levels of metabolites across species and is therefore directly comparable among metabolites despite lack of multispecies data on absolute concentrations. We note that similar phylogenetic inference methods have been widely adopted to study gene expression evolution (19, 20).

MCS displays extensive variation across metabolites in all four organs, spanning 560-fold to 970-fold ranges (Fig. 1*B*). Importantly, variation in MCS is not caused by technical artifacts, as within-species variation and measurement noise explain less than ~7% of total variance in MCS (*SI Appendix*, Fig. S2, *Methods*) and using an evolutionary model that explicitly incorporates such variation results in highly similar MCS values (*SI Appendix*, Fig. S3 and Dataset S1A, *Methods*). Phylogenetic comparisons of metabolomes might be confounded by dietary and environmental differences between species (6). To address this issue, we additionally analyzed a metabolome dataset of fibroblasts isolated from 16 mammals and cultured under identical in vitro conditions (21). Despite large differences between cell types and study conditions, the MCS values calculated from the in vitro fibroblast data show highly significant correlations with those based on the in vivo assessment of the four organs (*SI Appendix*, Fig. S4 and Dataset S1A). Furthermore, MCS extensively varies among different metabolites (up to 290-fold) in the fibroblast dataset as well. Thus, large differences in the conservation of individual metabolite concentrations hold in an independent dataset that is not confounded by environmental differences. This, together with previous observations in primates (6), suggests that environmental differences do not substantially confound phylogenetic comparisons of tissue metabolomes.

Conservation of a metabolite might vary across the phylogeny due to lineage-specific shifts in selective pressure (i.e., adaptive evolution). However, such heterogeneities are unlikely to confound our inferences. First, in each organ, only a small fraction (7 to 29%) of metabolite levels show lineage-specific shifts (7), and MCS is robust to such effects (*SI Appendix*, Fig. S5, see *Methods*). Second, we show that the rate of evolutionary change of metabolite levels is largely homogenous across the phylogenetic tree. To assess the variability of the rate of evolutionary change between clades, we calculated separate MCS values on two independent clades of the tree: rodents, rabbit and primates versus all other placental mammals (Dataset S1A). These two clade-specific MCSs strongly correlate with each other in all organs (see Fig. 1*C* for liver and *SI Appendix*, Fig. S6 for other organs), indicating that metabolites that are conserved in a particular clade also tend to be conserved in the rest of mammals.

Together, these results indicate that the evolution of individual metabolite levels can be characterized by a simple conservation score, which is largely invariant across mammalian clades, but varies extensively across metabolites.

### Abundance and Essentiality Are Important Determinants of Evolutionary Conservation.

**Fig. 2.**
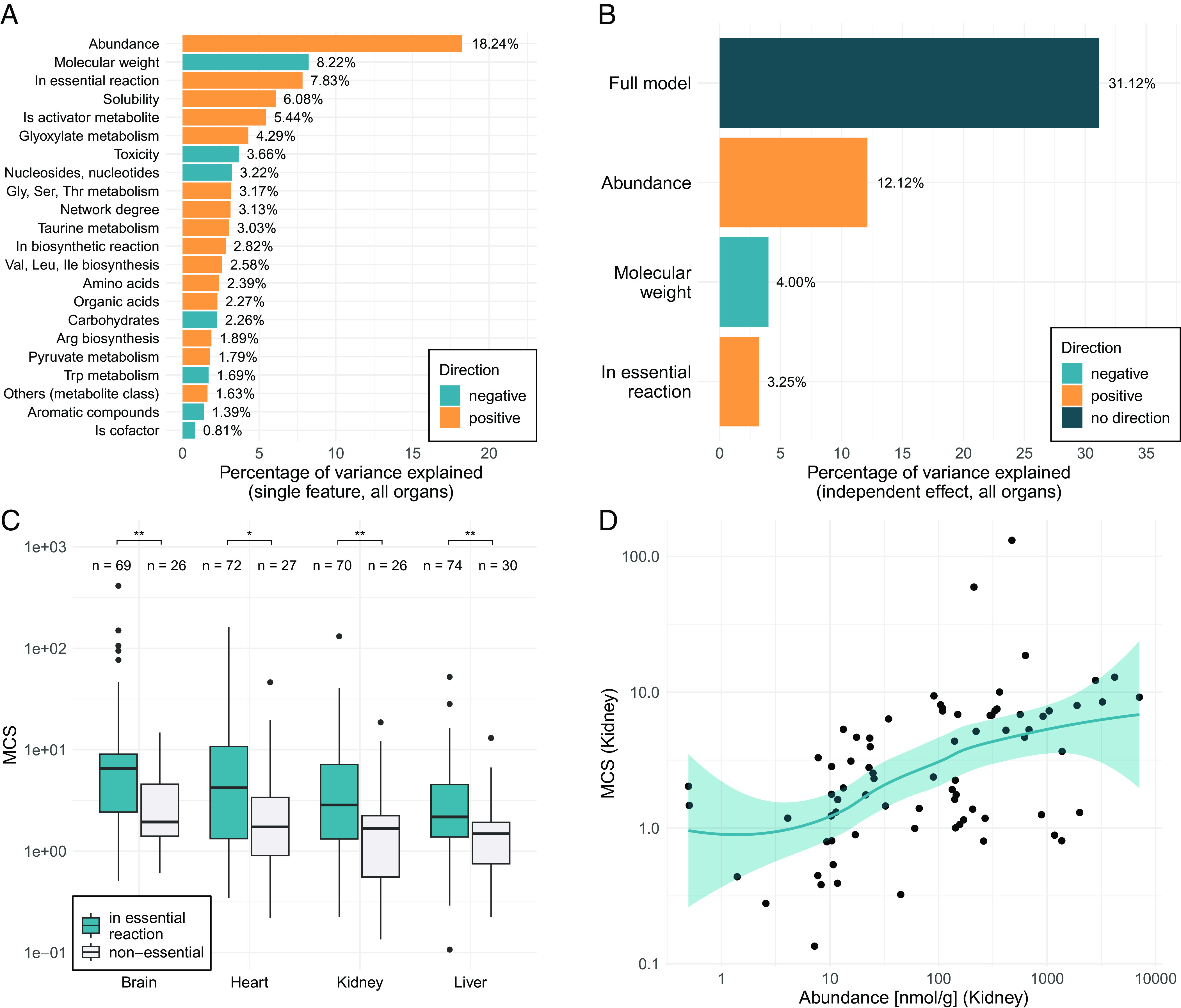
Determinants of MCS. (*A*) Barplot showing the percentage of variance in conservation score explained by each metabolite feature individually (i.e., univariate models). Only features with a significant effect are shown (false discovery rate adjusted p-value < 0.01, *Methods*). (*B*) Barplot showing the percentage of variance in conservation score explained by multivariate regression modeling. Bars indicate the percentage of variance explained by the full model, as well as the independent contribution of each feature found to be significant in the model (*P* < 0.05). (*C*) Metabolites participating in reactions encoded by essential genes show higher conservation scores than the rest of metabolites (two-sided Wilcoxon tests, brain: *P* = 0.0013; heart: *P* = 0.019; kidney *P* = 0.0049; liver *P* = 0.0022). Boxplots show the median, first and third quartiles, with the whiskers showing the values within a 1.5 interquartile range distance from the first and third quartiles. (*D*) Metabolites with high absolute concentration (abundance) show higher conservation scores (kidney: Spearman’s correlation rho = 0.53, *P* = 1.1e−06, N = 76; for other organs see *SI Appendix*, Fig. S7). Line indicates LOESS regression, with their 95% CI indicated in blue.

By analogy to the neutral theory of sequence evolution (22), we hypothesize that conserved metabolites are subject to stronger functional constraints against concentration changes. If so, MCS should largely be determined by the functional role and biochemical properties of metabolites. To test this, we compiled 17 features capturing the biological and chemical properties of metabolites, including pathway membership (Dataset S1B), the number of network connections (network degree), absolute metabolite concentration (abundance) in mice (17), chemical class, toxicity, and various physicochemical properties (Fig. 2*A* and *SI Appendix*, Table S1 and Dataset S1A, see *Methods*). We also included “essentiality,” a feature describing whether a metabolite participates in a reaction catalyzed by an essential enzyme, as determined by gene deletions in mice (23).

We found several metabolite features that are statistically significantly associated with MCS when analyzed individually (Fig. 2*A* and *SI Appendix*, Table S1, *Methods*). As metabolite features might correlate with each other, we next carried out multivariate regression to identify independent predictors of MCS by jointly analysing the four organs (*Methods*). We found that a simple model with three dominant predictors explains ~31% of variation in MCS across metabolites (Fig. 2*B* and *SI Appendix*, Table S3). This is a remarkably high figure when considering that we estimate the upper limit of the predictability of MCS at 49%, based on its reproducibility between two independent clades (Fig. 1*C* and *SI Appendix*, Table S2). Notably, the portion of variance explained in metabolite conservation is comparable to the explained evolutionary rate variation in protein sequences (~40%) (24).

Three general metabolite properties have significant independent effects on MCS in all organs: abundance, molecular weight, and involvement in essential reactions (Fig. 2*B* and *SI Appendix*, Table S4). Specifically, metabolites that are i) highly abundant in the cell, ii) have a small molecular size or iii) participate in reactions encoded by essential genes tend to be more conserved (Fig. 2 *C* and *D*). The latter finding is consistent with the intuitive expectation that perturbations in metabolites connected with essential enzymes are especially harmful, and are therefore subject to stronger stabilizing selection.

Abundance is the strongest determinant of conservation in all organs (Fig. 2*B* and *SI Appendix*, Table S4). Metabolites vastly differ in their absolute concentrations, spanning over six orders of magnitude (10). We revealed that higher abundance of a metabolite is associated with a higher conservation score, with a continuous trend across the entire range of abundance (Fig. 2*D* and *SI Appendix*, Fig. S7). We emphasize that abundance data come from an independent study that quantified absolute metabolite concentrations for several matching organs in mice (17). Low-abundance metabolites are typically measured with larger noise, potentially underestimating their conservation scores. However, such a bias is unlikely to confound our results because the positive correlation between abundance and MCS remains highly similar when explicitly accounting for measurement variability in the conservation score calculations (*SI Appendix*, Table S5 versus *SI Appendix*, Fig. S7, *Methods*). Furthermore, the conclusion also holds when inferring MCS from the in vitro fibroblast dataset and correlating it with absolute metabolite concentrations measured in mouse cell culture (10), suggesting that it is not an artifact of comparing species with different diets (*SI Appendix*, Fig. S8).

Conservation is largely independent of the chemical class, physicochemical properties, toxicity, pathway membership, network position, and interaction degree of metabolites in the multivariate model (*SI Appendix*, Table S4). For instance, while metabolites participating in many reactions or serving as allosteric activators of enzymes tend to be conserved, these relationships disappear when accounting for other metabolite properties (*SI Appendix*, Table S4). Thus, contrary to intuitive expectations, metabolites interacting with multiple enzymes are not more strongly constrained. Furthermore, the toxic effects of highly increased metabolite levels, as assessed by toxicity in mice (25), do not appear to constrain metabolome evolution.

Together, these findings demonstrate that evolutionary conservation of metabolite levels is well predictable based on a handful of metabolite properties, with abundance being the primary determinant.

### Organ-Specific Metabolite Conservation Reflects Differential Functional Constraints.

We next sought to investigate the differences in individual MCSs between different organs. We hypothesize that such shifts in conservation arise from organ-specific biological functions.

**Fig. 3.**
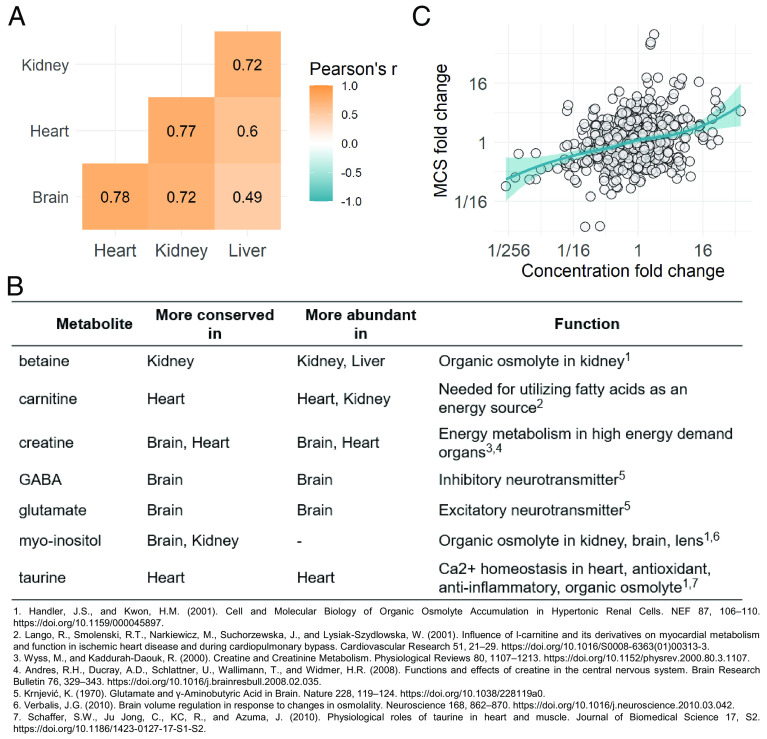
Organ-specific differences in MCS. (*A*) Similarity of conservation scores of metabolites across the four organs as measured by Pearson’s correlation coefficients. All organ comparisons are highly significant (*P* < 1.1e^−10^, N = 113 to 122). (*B*) Examples of shifts in metabolite conservation among organs that likely reflect organ-specific functions. Table shows organ-specific conservation and abundance patterns of selected metabolites (i.e., indicating the organs in which a specific metabolite is more conserved or abundant relative to the cross-organ average) and a description of their organ-specific functions. (*C*) Between-organ differences in metabolite levels show a positive correlation with between-organ conservation score differences (Spearman’s rho = 0.27, *P* < 10^−4^ from permutation test). For each metabolite, metabolite-level difference (fold-change) and conservation score difference (fold-change) were calculated for all six possible organ pairs. Each dot represents the comparison of two organs for a particular metabolite. Line indicates LOESS regression. Statistical significance was assessed by permutation (*Methods*).

In general, MCS correlates well among the four organs, indicating similar amounts of metabolite-specific functional constraints across organs (Fig. 3*A*). This is consistent with our finding that a handful of metabolite properties universally determine conservation in all four organs (Fig. 2*B*). Nevertheless, some metabolites are much more conserved in one organ than in the others (*SI Appendix*, Table S6). Literature data suggest that such differences partly reflect organ-specific metabolite functions (Fig. 3*B*). For example, both gamma-aminobutyrate and glutamate show the strongest conservation in the brain, where they serve as the principal inhibitory and excitatory neurotransmitters, respectively (26). Similarly, the osmolytes betaine and myo-inositol are especially conserved in the kidney and such molecules have key roles in protecting renal medullary cells from high NaCl and urea levels (27).

Several metabolites display elevated levels in specific organs, independent of the species (7). Given that abundant metabolites tend to show enhanced conservation within organs (Fig. 2*D*), we hypothesize that a particular metabolite should be more conserved in organs where it is more abundant. Indeed, several metabolites displaying organ-specific conservation also have higher levels in those organs where they are more conserved (Fig. 3*B*). As a systematic test, we examined the relationship between the relative differences (fold-change) of metabolite levels and conservation scores among different organs (Fig. 3*C*). As expected, between-organ differences in metabolite levels and between-organ conservation score differences display a significant positive correlation (Fig. 3*C*). Note that since the total amount of evolutionary changes in the metabolome varies by organ (7), we compared the conservation of individual metabolites across organs while accounting for this metabolome-wide effect (*Methods*).

Together, these results indicate that metabolites vary in their conservation due to differing amounts of functional constraints, partly reflecting organ-specific metabolite functions, and highlight the key influence of abundance on metabolite conservation.

### Systems Modeling Illuminates the Mechanism of Functional Constraint.

Why are the metabolites that are abundant or involved in essential reactions highly conserved? Metabolite concentrations are principal determinants of reaction rates (fluxes) in the network (3). As metabolic fluxes obey optimality principles (28), we propose that selection to maintain key metabolic fluxes at optimal values constrains the evolution of metabolite levels, and may explain the higher conservation of metabolites that are abundant or involved in essential reactions. To test this, we simulated evolution in a physiologically relevant mathematical model of central metabolism. We employed a kinetic model of the core metabolism of human erythrocytes, which includes glycolysis, the 2,3-bisphosphoglycerate shunt and the pentose-phosphate cycle, with 40 internal metabolites (29). The model allowed us to simulate the effects of changes in enzyme kinetic parameters (i.e., mutations) on steady-state fluxes and metabolite concentrations (*Methods*). Assuming that the nonmutated model represents a fitness maximum resulting from past evolutionary optimization of erythrocyte metabolism, we approximated the deleterious effect of mutations by calculating deviations in four specific fluxes, referred to as key fluxes, that are important for proper erythrocyte functioning (29) (*Methods*). Then, we simulated evolution with and without stabilizing selection to maintain the levels of these key fluxes, the latter corresponding to evolution under pure genetic drift, using a Markov chain Monte Carlo (MCMC) approach (30) (*SI Appendix*, Fig. S9, *Methods*). In the pure genetic drift scenario, we accepted all mutations that led to a steady-state solution, while in the stabilizing selection scenario we only accepted those mutations that had only a very minor effect on the key fluxes. Specifically, we applied selection to maintain key fluxes around their wild-type values by removing those mutations that altered these fluxes beyond a predefined threshold (*Methods*). As a consequence, the metabolome differences accumulated over many iterations are neutral because the key fluxes in the network still take near wild-type values. Thus, these scenarios represent nonadaptive–neutral–modes of evolution.

**Fig. 4.**
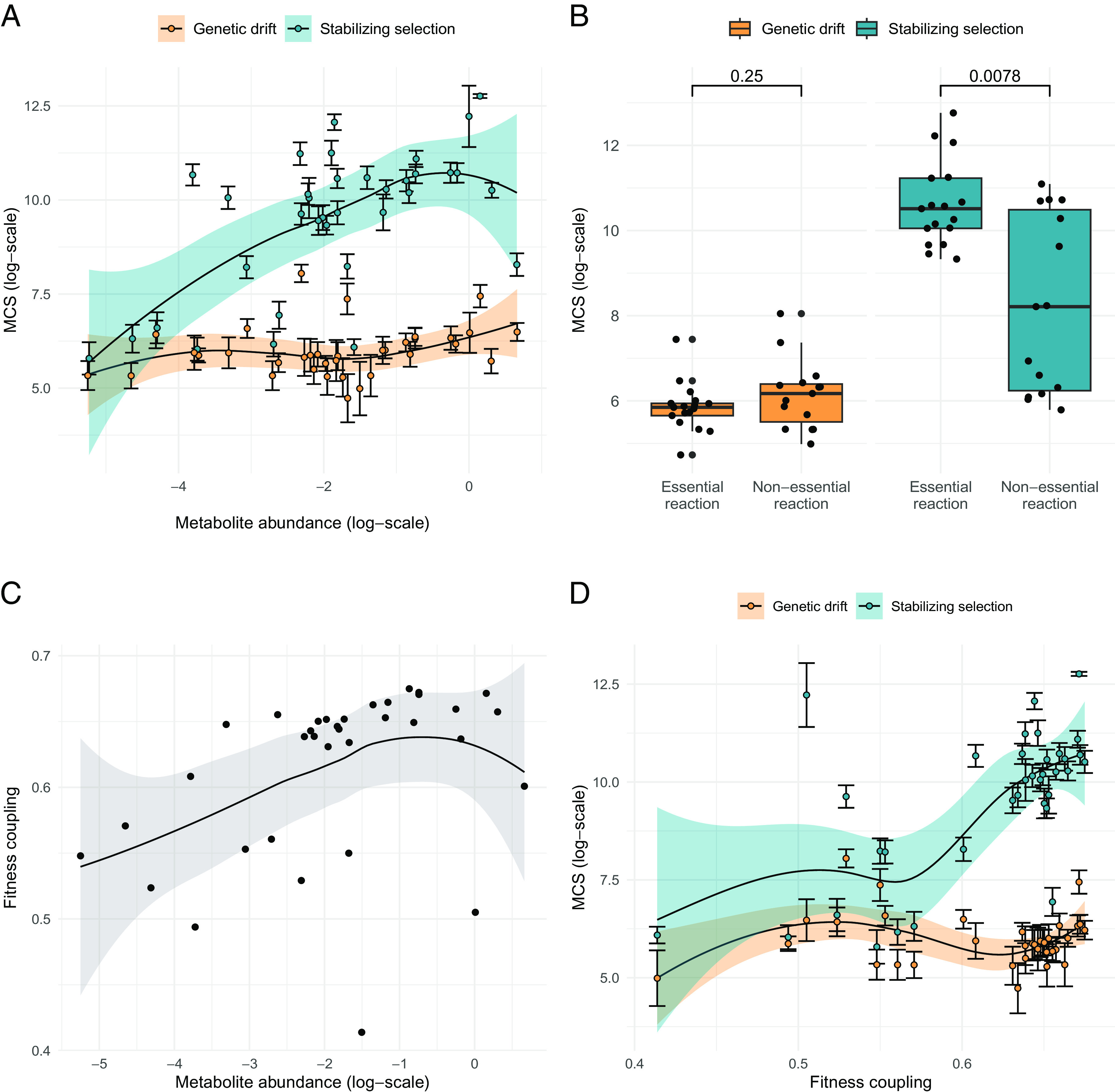
Functional constraints in an in silico model of metabolic evolution. (*A*) Metabolites with a higher abundance in the wild-type model show higher conservation scores in the presence of stabilizing selection (blue dots, Spearman rho = 0.55, *P* = 6.63e−4, N = 35), but not in the absence of selection (orange dots, Spearman rho = 0.30, *P* = 0.078, N = 35). Each dot and error bar represents the mean and SD of the MCS, calculated for a particular metabolite based on 10 simulations. (*B*) Metabolites involved in essential reactions (i.e., their products or substrates) have higher conservation scores than those involved in nonessential reactions in the presence of stabilizing selection (blue), but not in the absence of selection (orange), as indicated by two-sided Wilcoxon tests (*P* = 7.82e−3 and *P* = 0.246, respectively). Each dot represents the mean conservation score for a particular metabolite based on 10 simulations. (*C*) Wild-type abundance of metabolites correlates with their extent of fitness coupling (Spearman rho = 0.49, *P* = 3.14e−3, N = 35). (*D*) MCSs correlate with the extent of fitness coupling under stabilizing selection (Spearman rho = 0.55, *P* = 7.78e−4, N = 35), but not in the absence of it (Spearman rho = 0.06, *P* = 0.724, N = 35). The lines in panels *A*, *C*, and *D* represent LOESS regressions, with their 95% CIs shown. The boxplots in panel *B* show the median, first, and third quartiles, with the whiskers showing the values within a 1.5 interquartile range distance from the first and third quartiles.

As expected, in silico MCSs are much higher in the presence of stabilizing selection (*SI Appendix*, Fig. S10), demonstrating that many metabolome-altering mutations are harmful. Furthermore, between-metabolite differences in MCS increase significantly under stabilizing selection (*SI Appendix*, Fig. S10), indicating that the requirement to maintain key fluxes imposes varying levels of constraint across different metabolites. Remarkably, metabolite abundance and involvement in essential reactions are determinants of in silico MCS. In particular, we found a significant positive correlation between a metabolite’s abundance in the wild-type (i.e., nonmutated) model and its conservation score in the simulations (Fig. 4*A*). Similarly, metabolites involved in reactions that are deemed essential in silico (i.e., reactions that have a large impact on key fluxes when inactivated) show high conservation scores in the simulations (Fig. 4*B*, *Methods*). Importantly, these associations hold only in the presence of stabilizing selection, indicating that they are not caused by mutational variability (Fig. 4 *A* and *B*). Furthermore, a multivariate analysis indicates that metabolite abundance and reaction essentiality are independently associated with in silico MCS (*SI Appendix*, Table S7).

We next hypothesized that mutations altering abundant metabolites are more likely to perturb key fluxes and are therefore selected against. To test this, we defined a measure of fitness coupling for each metabolite by simulating the impact of single mutations and calculating how strongly changes in the levels of each metabolite are correlated with changes in key fluxes (*Methods*). Consistent with the hypothesis, abundant metabolites are indeed more strongly coupled to fitness (Fig. 4*C*). Furthermore, the extent of fitness coupling of a metabolite correlates with its conservation score inferred under stabilizing selection, but not under pure genetic drift (Fig. 4*D*). In addition, the four key fluxes assumed to be important for fitness (29) lead to a significantly stronger correlation between metabolite abundance and fitness coupling than randomly defined key fluxes (*SI Appendix*, Fig. S11*A*; *Methods*). Thus, the strong coupling of highly abundant metabolites to fitness is not a by-product of the modeling procedure, but specifically holds for a fitness definition that captures the biochemical functions of the erythrocyte metabolic network (29). Further analysis confirmed that the strong conservation of abundant metabolites is mediated by fitness coupling (*SI Appendix*, Fig. S11*B*).

Finally, the above results also hold for a different model of erythrocytes and a model of human hepatic glucose metabolism (*SI Appendix*, *Appendix* S1). Overall, these findings indicate that metabolites that are highly abundant or participate in essential reactions are more conserved in their levels because they are more crucial to maintain key metabolic fluxes. Importantly, as beneficial mutations are not included in the simulations, all accumulated metabolome differences are neutral. Thus, we conclude that a simple neutral model of metabolome evolution is sufficient to explain major empirical patterns of metabolome conservation.

### Evolutionary Conservation Informs on Disease Association.

**Fig. 5.**
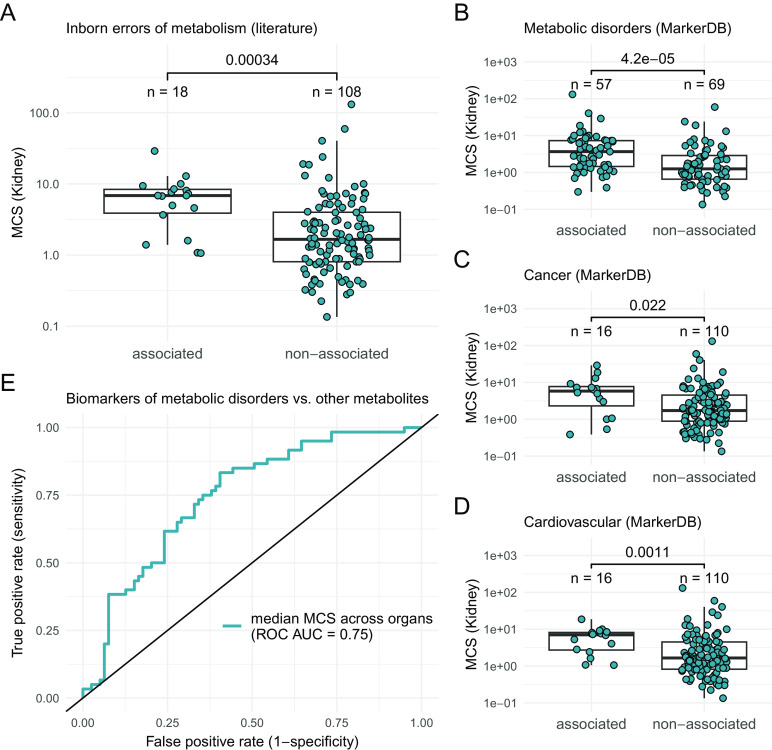
Levels of disease-associated metabolites are highly conserved. (*A*) Metabolites involved in IEM show significantly higher conservation scores than the rest of metabolites in the kidney (two-sided Wilcoxon rank sum test). For other organs, see *SI Appendix*, Fig. S12. (*B*–*D*) Metabolites associated with three broad disease conditions (metabolic disorders, cancer, and cardiovascular disorders) show a significantly higher level of conservation than the rest of metabolites in kidney (*P*-values from the Wilcoxon rank sum tests are shown in figure). Boxplots show the median, first and third quartiles, with the whiskers indicating the values within a 1.5 interquartile range distance from the first and third quartiles. (*E*) ROC for prediction of biomarkers of metabolic disorders based on the median conservation score across organs.

Next, we asked whether the conservation of a metabolite informs on its association with diseases. We first focused on human inborn errors of metabolism (IEMs), which are genetic disorders caused by disruption of specific metabolic pathways (31). The early onset and high severity of these disorders suggest that metabolites associated with IEMs might be highly constrained in mammals. We compiled metabolites known to be involved in the disease etiology or the diagnosis of IEMs routinely measured in newborn screening (*Methods*, *SI Appendix*, Table S8). We found that IEM-associated metabolites show significantly higher conservation scores than the rest of metabolites in all four organs (Fig. 5*A* and *SI Appendix*, Fig. S12). The strong evolutionary conservation of IEM-associated metabolites is not explained by abundance, a particular class of conserved metabolites or specific metabolic pathways (*SI Appendix*, Table S9), suggesting that it reflects their importance for normal metabolic functioning.

To test whether this finding applies to other diseases beyond IEM, we used MarkerDB, a comprehensive database of clinical biomarkers (32) and focused on 11 broad disease conditions with sufficient numbers of metabolites (*SI Appendix*, Table S10 and Dataset S1C). As expected, metabolites associated with metabolic disorders, including many IEMs, tend to be highly conserved (Fig. 5*B*). More remarkably, we identified two additional broad disease conditions—cancers and cardiovascular diseases—that are independently associated with highly conserved metabolites (Fig. 5 *C* and *D* and *SI Appendix*, Table S10, *Methods*). For instance, choline, a precursor in lipid metabolism, is highly conserved in all four organs, but is not associated with any IEMs (*SI Appendix*, Fig. S13). Notably, abnormal choline metabolism is a general hallmark of cancers, and both phosphocholine and total choline-containing metabolite levels are used to detect malignant tumors (33). Furthermore, the oncometabolite succinate (34) also shows marked conservation in several organs (*SI Appendix*, Fig. S13).

Biomarkers of metabolic disorders show the strongest signal of conservation (Fig. 5*B*), suggesting that evolutionary conservation could potentially be leveraged to identify such biomarkers independently of previous clinical knowledge. As a preliminary test, we computed an aggregate score of conservation across the four organs (*Methods*). Encouragingly, this conservation score alone separates biomarkers of metabolic disorders from the rest of metabolites with reasonable accuracy (area under the ROC curve = 0.75, Fig. 5*E*).

Finally, we hypothesized that metabolites involved in multiple diseases are more likely to affect organismal fitness when altered and hence are under stronger stabilizing selection. Indeed, the conservation score shows a positive correlation with the number of specific diseases involving a particular metabolite (*SI Appendix*, Fig. S14; effect is independent of abundance and essentiality, *SI Appendix*, Table S11).

### Metabolome Conservation in the *Drosophila* Genus.

To test the generality of our main findings, we also analyzed metabolome evolution in the distantly related genus *Drosophila*, across a 50-My phylogeny (*SI Appendix*, Fig. S15*A*). We calculated metabolite-specific conservation scores using data on 92 nonlipid metabolites measured in whole adults of 11 *Drosophila* species under the same controlled environment (35) (Dataset S1D, *Methods*). Just as in mammals, MCS varies extensively across different metabolites, spanning over three orders of magnitude (*SI Appendix*, Fig. S15*B*). Remarkably, using an independent dataset of absolute metabolite concentrations in *Drosophila melanogaster* (36) (Dataset S1D), we found a significant positive correlation between abundance and the conservation score, with an effect size similar to that in mammals (*SI Appendix*, Fig. S15*C*). Furthermore, metabolites involved in human IEMs tend be highly conserved in *Drosophila* as well (*SI Appendix*, Fig. S15*D*). We conclude that metabolome evolution is governed by similar principles in two distant animal phyla.

## Discussion

In this work, we combine phylogenetic analysis of metabolome data with systems biology modeling to seek general principles governing the evolution of the levels of tissue metabolites in animals. By introducing a measure of evolutionary conservation of individual metabolite levels, we showed that the extent of conservation of a given metabolite is largely invariant between closely related clades, but varies extensively across metabolites. Such variation in conservation is predictable based on a few metabolite properties and is consistent with a simple model where natural selection preserves flux through key metabolic reactions while permitting the accumulation of selectively neutral changes in enzyme activities. We further demonstrated that this general conceptual framework of metabolome conservation informs on disease associations and biomarker status of metabolites.

Metabolite abundance emerged as the main determinant of conservation, with highly abundant metabolites displaying the highest level of conservation, as evidenced by three independent datasets of absolute metabolite concentrations in mice (Fig. 2), *D. melanogaster* (*SI Appendix*, Fig. S15) and in vitro cell culture (*SI Appendix*, Fig. S8). Importantly, any particular metabolite displays stronger conservation in organs where it is more abundant, demonstrating that abundance per se affects conservation. Systems modeling further showed that abundant metabolites are subject to stronger functional constraints (Fig. 4). Why should it be so? First, the levels of highly abundant metabolites might be more rate-limiting for key fluxes than those of low-abundance metabolites, implying a causal effect of metabolite level on fitness. Specifically, it might be the case that metabolic systems have evolved toward optimal states where the concentrations of abundant metabolites have lower margins of safety around their optimal values, analogous to the “expression cost” hypothesis to explain the elevated sequence conservation of highly expressed proteins (14, 37). This argument assumes that the optimal level of a metabolite reflects a trade-off between the benefits (e.g., enzymatic rate) and cellular costs of metabolite production. Under such optimal conditions, the benefit and cost of having an extra molecule should be equal and identical across metabolites (i.e., have the same marginal values). As a consequence, a mutation that reduces the level of a metabolite by a given fraction would cause a larger loss of benefit for a highly abundant metabolite than for a lowly abundant one. This would be analogous to the observation that halving gene dosage is generally more deleterious for highly expressed genes (37). Alternatively, abundant metabolites might not be particularly rate-limiting, but might be subject to stronger indirect selection due to the harmful side effects of mutations affecting their levels (38). We speculate that there might be less ways to alter abundant metabolites without also perturbing key fluxes, resulting in stronger indirect selection on these molecules. Finally, regardless of their specific functions, abundant metabolites incur broad cellular costs due to limitations on osmotic pressure (12) and total dry mass (11), potentially constraining their evolution. Clearly, further studies are needed to test these scenarios.

Our work has profound implications for the neutral theory of molecular evolution, which posits that most within- and between-species variations at the molecular level are selectively neutral rather than adaptive (22). While the theory explains many aspects of sequence and gene expression evolution (20, 22, 39), it has been unclear whether it applies to variations at the metabolome level, which is more closely related to phenotypes (40). Our results are broadly consistent with a neutral model of metabolome evolution. First, MCSs are largely constant across different mammalian clades, suggesting that similar evolutionary forces shape the metabolome despite extensive phenotypic divergence. Second, conservation is determined by the functional properties of metabolites, namely abundance, involvement in essential reactions and association with human diseases. As these metabolite properties likely reflect the level of functional constraints, rather than the amount of adaptive evolution, they support the neutral model. Notably, analogous gene properties—expression level, essentiality and disease association (13)—determine protein sequence conservation, revealing a striking parallel between the selective constraints driving metabolome and protein evolution. Finally, metabolic modeling demonstrated that stabilizing selection on key fluxes is sufficient to explain the strong conservation of abundant and essential metabolites without the need to invoke adaptation to changing environments. Specifically, the model simulations included only neutral and deleterious mutations, but not beneficial ones, yet recapitulated the main empirical observations. Together, these results suggest that a substantial fraction of metabolome differences among mammals, as well as among *Drosophila* species, are neutral and are permitted rather than favored by selection. It remains to be tested whether further predictions of the neutral model are fulfilled, and whether they also hold for other major taxa. Nevertheless, our simulation study represents an important step toward a theoretical framework of metabolic evolution driven by nonadaptive processes.

The evolutionary history of gene sequences and gene expression levels informs on their disease involvement (16, 20). Our work expands this notion to include an additional layer of molecular phenotypes by showing that metabolome conservation is predictive of the disease associations of metabolites. Remarkably, biomarkers can be distinguished from nonbiomarker metabolites simply based on the comparison of metabolomes across species, without utilizing any prior clinical knowledge. As expected, metabolite conservation appears to be most informative for metabolic diseases that disrupt basic cellular functions and show an early onset, such as inborn errors of metabolism. More intriguingly, metabolites associated with tumorigenesis are also well conserved, suggesting that cancer avoidance might be an important selective force in wild mammals (41).

We emphasize that while some metabolite biomarkers are causally involved in disease development, the exact nature of many metabolite–disease associations remains unclear. In some cases, metabolite dysregulation could very well be a consequence of the disease itself, while in others it could precede any symptoms and even be an indicator of future disease onset. We note that elevated evolutionary conservation can result either from direct selection on a disease-causing metabolite or indirect (apparent) selection on a metabolite that correlates with disease states. Therefore, we expect that large alterations in the levels of metabolites that are otherwise subject to strict evolutionary constraints may indicate underlying health issues even if there is no causal relationship between the metabolite and the disease.

We anticipate that evolutionary metabolomics should have at least two possible applications to aid clinical diagnosis. First, it offers a strategy to identify metabolites whose dysregulation matters the most to human health and therefore could be involved in disease mechanisms or may be used as biomarkers. Given the plethora of assayed metabolites in metabolomic epidemiology studies (42), evolutionary conservation may help to prioritize them for biomarker identification and further investigations. Second, it might be possible to infer the range of permissible metabolite levels from cross-species data, and use this information to detect pathogenic alterations in individual metabolome profiles (20). As clinical diagnoses typically rely on the measurement of plasma metabolite levels, it is an important open issue whether the concept of evolutionary conservation could be applied to blood metabolomes, which might be more strongly influenced by environmental effects than tissue metabolomes.

In sum, our findings illustrate how evolutionary comparisons of metabolite levels on a network scale can be leveraged to study the functional constraints and pathogenic alterations of cellular metabolism.

## Methods

### Calculating MCSs.

To study metabolome conservation in mammals, we obtained metabolomic measurements of 139 nonlipid metabolites from a multispecies study (7). The dataset contains relative metabolite levels across 26 mammalian species in four organs (brain, heart, kidney, and liver) and is based on targeted metabolomics measurements involving three distinct liquid chromatography–mass spectrometry (LC-MS) methods. Note that the measured samples were homogenates of freshly frozen tissues of killed animals, matched by age (i.e., young adults) and sex (7). We calculated the mean of the normalized (log_10_-transformed) relative metabolite level across all biological replicates so that each metabolite in each species and organ is represented by a single relative concentration value. We then used these values as continuous molecular traits for which conservation scores are computed.

To calculate the MCS of individual metabolites, we first fit a simple BM model of trait evolution on the relative levels of each metabolite in each organ across the phylogeny, using the fitContinuous function in the Geiger R package (43).

The BM model represents evolution of a continuous trait through time as a random walk process in which, during any elapsed time Δt, the value of the trait ( x ) changes by a random number drawn from a normal distribution with a mean of 0 and a variance of σ^2^Δt. As such, at any timepoint t_1_, the level of the trait can be estimated as:

[1] $$x\left(t_{1}\right)\;∼\;N\left(x\left(t_{0}\right),\sigma^{2}\mathrm{\Delta t}\right).$$

This BM process can then be applied to a phylogenetic tree, as described by Felsentein (44). In short, given a known phylogeny and known trait values at the tips of the tree, we can use Felsenstein’s method to estimate the value of σ^2^ that gives rise to the observed trait values in the timeframe supplied by the branch lengths of the phylogenetic tree, while also accounting for the phylogenetic relationships between the species. The evolutionary rate parameter of the BM model, σ^2^, measures the rate of trait diversification along the phylogeny and is in the units of trait variance increase per unit evolutionary time (as approximated by phylogenetic distance).

It has been argued that the rate parameter of a simple BM model is a useful measure of the “effective rate” of trait evolution, even if more complex evolutionary models fit a given trait better (18). Next, by taking the inverse of the evolutionary rate parameter, we define a measure of metabolite conservation, where metabolites that diverge more slowly in their levels over time are represented by higher conservation scores. Note that comparison of MCS scores across metabolites does not demand direct comparisons of concentrations between metabolites and hence no data on absolute concentrations are required.

Conservation scores were calculated in a similar fashion for relative metabolite levels measured in fibroblast cell cultures (21) and *Drosophila* species (35) as well. The phylogenetic trees used in these calculations were obtained from refs. 21 and 35 and from http://www.timetree.org/ (45).

### Variation of Conservation Scores among Different Metabolites versus Biological Replicate Measurements.

To assess the impact of measurement noise and/or within-species variation on the inferred MCSs and compare it to among-metabolite variation, we made use of multiple biological replicate measurements. Specifically, we sampled randomly with replacement concentration values from two to four biological replicate measurements, depending on the species and the organ, and re-calculated MCSs 100 times (i.e., bootstrap procedure). For each organ, metabolite/species pairs having only one replicate were removed from the analysis (89.1% of all organ—species–metabolite triplets have more than one replicate). For each organ, we then applied a one-way ANOVA test on the resulting MCS distributions to partition the amount of total variance in conservation scores into between-metabolites variance and error variance, the latter capturing variation between biological replicate measurements of the same metabolites.

### Metabolite Features Associated with MCS.

Seventeen distinct classes of metabolic features were collected in order to probe their relationship with MCS. Information on the regulatory roles of metabolites (enzyme activator, inhibitor, and cofactor function) was obtained from ref. 46 which was compiled from the BRENDA database (47). Information on the chemical properties of metabolites (chemical class, molecular weight, dissociation constants, water solubility, and hydrophobicity) were collected from the HMDB and KEGG databases (48, 49). Metabolite toxicity information, in the form of mouse LD50 values (the concentration of the metabolite that is lethal to 50% of specimens, in mg/kg) was collected from the ChemIDPlus database (https://chem.nlm.nih.gov/chemidplus/). Pathway membership and broad position in the metabolic network (biosynthetic, degradation, and energy metabolism) were collected from the KEGG and HumanCyc databases, respectively (48, 50). We note that pathway membership was used only for those metabolic pathways that contained at least five metabolites for which we had conservation score calculated, yielding 27 pathways in total. Organ-specific absolute metabolic abundance measurements were obtained for mouse from the Mouse Multiple tissue Metabolome DataBase (MMMDB) (17). Network degree (the number of reactions a metabolite participates in) was determined using a genome-scale reconstruction of the human metabolic network (51). Metabolites involved in metabolic reactions that are encoded by essential genes were identified using phenotypic data from mouse knockout lines. In short, we identified genes whose deletion caused either a lethal phenotype or infertility in the Mouse Genome Database (23). Next, we defined essential reactions as reactions where the majority (>50%) of the genes associated with the reaction in the human metabolic network are essential. A metabolite was considered part of the essential set if it participates in at least one essential reaction. For brevity, we refer to such metabolites as “essential” metabolites, even though metabolite essentiality cannot be directly measured.

To probe the relationships between MCS and individual metabolite features, we used linear regression modeling. For each individual feature, we fitted a linear model that accounts for both the effect of the given feature and the organ in which the conservation scores were estimated (i.e., organ membership). This allowed us to assess the general effect of each feature on evolutionary conservation across all four organs simultaneously, while accounting for organ-specific global differences in conservation score. The percentage of variance in MCS explained by each feature (as shown in Fig. 2*A* and *SI Appendix*, Table. S1) was calculated by subtracting the R^2^ value of a model containing organ-membership as the only predictor variable from the R^2^ value of the model containing both organ-membership and the feature of interest as predictor variables. Each chemical class and KEGG pathway was evaluated separately.

To identify the main determinants of evolutionary conservation while controlling for potential covariations between metabolite features, we performed a multivariate analysis as follows. We fitted an initial linear model that included all features and metabolic pathways that individually had a significant effect on conservation scores (nine metabolic features and seven specific metabolic pathways, as shown in *SI Appendix*, Table S4). Next, we used a stepwise feature selection (using the step function in R) to identify the most parsimonious linear model that contains the combination of features that provides the best fit based on the Akaike information criterion. To quantify the contribution of the individual metabolite features to the most parsimonious model (i.e., independent effect), we fitted simpler models by leaving out single features and calculating the decrease in the adjusted R^2^ value. The portion of variance in MCS explained by the combination of metabolite features was determined by subtracting the independent effect of organ membership from the adjusted R^2^ value of the most parsimonious multivariate model. Note that, in order to minimize the number of features in the multivariate model, we used chemical class as a single multilevel factor, instead of multiple binary features.

### Between-Organ Differences in MCS.

For all between-organ analyses, we only included metabolites that were measured in all four organs (110 metabolites of a total of 139). To compare the conservation of metabolites across organs, we first normalized the conservation scores as two of the four organs, brain and heart, are generally more strongly conserved than the others (i.e., show smaller amounts of total evolutionary divergence across the whole metabolome). Conservation scores were first normalized by log_2_ transformation and then centered on zero for each organ. Then, we calculated the organ-specific deviation in conservation for each metabolite by taking the normalized conservation score from one organ and subtracting the mean normalized scores of the other three organs from it. High conservation deviation for a given organ indicates that the metabolite is more strongly conserved in that particular organ compared to other organs. For each organ, we identified the top 10% most strongly deviating metabolite (*SI Appendix*, Table S6).

To test whether between-organ differences in metabolite levels are generally associated with shifts in MCSs, we first calculated, for each metabolite, the differences in the normalized conservation scores between all organ pairs. Next, between-organ differences in metabolite levels were determined by calculating the log_2_ fold change of metabolite levels between all organ pairs for each species and then taking the average of the species-specific fold change values. Thus, each metabolite is described by six conservation scores and six metabolite-level fold change values, corresponding to all six possible comparisons among the four organs. We then quantified the association between all metabolite-level fold change and conservation score fold change values across all metabolites using the Spearman’s rank correlation coefficient. Because the fold change values associated with a given metabolite are not independent from each other, we calculated the *P*-value of the correlation using a permutation test as follows. We randomly reassigned the organ memberships of MCSs and recalculated the Spearman’s correlation coefficient across 10,000 permutations, to test whether the observed correlation is significantly higher than expected by chance (i.e., one-sided test).

### Evolutionary Simulations in a Mechanistic Model of Central Metabolism.

#### A kinetic model of the core metabolism of human erythrocytes.

We used a publicly available kinetic model of the human erythrocyte central metabolism, including glycolysis, the 2,3-bisphosphoglycerate shunt and the pentose-phosphate cycle (29). This model contains 40 variable metabolites, 38 kinetic reactions, and 166 kinetic parameters (http://jjj.biochem.sun.ac.za/models/holzhutter/). Four specific fluxes, referred to as key fluxes, are assumed to be important for the fitness: a) the formation of 2,3-bisphosphoglycerate (flux $\nu_{9}$ ), which modulates oxygen affinity of hemoglobin, b) ATP (adenosine triphosphate) utilization (flux $\nu_{16}$ ), which maintains Na/K gradients across plasma membrane, c) glutathione (GSH) oxidation (flux $\nu_{21}$ ), which prevents oxidative damage in the cell, and d) the synthesis of phosphoribosyl-pyrophosphate (flux $\nu_{26}$ ), required for the salvage of adenine nucleotides (29).

#### Calculating the fitness effect of mutations in the model.

The kinetic model allowed us to simulate the effects of changes in enzyme kinetic parameters (i.e., mutations) on steady-state fluxes and metabolite levels. Mutations are approximated by independent random perturbations to the parameters of the 38 kinetic equations. To simulate a single mutational event, one kinetic parameter p is selected at random (uniformly among reactions) and its mutant value p' is derived by multiplying it with a factor drawn from a log_10_-normal distribution of variance ${\sigma_{\mathrm{mut}}}^{2}$ (52):

[2] $$p'\to p\times 10^{\alpha },\alpha \;∼\;N\left(0,{\sigma_{\mathrm{mut}}}^{2}\right).$$

The mutational variance ${\sigma_{\mathrm{mut}}}^{2}$ is constant for all the kinetic parameters. The steady-state of each mutant model was computed using Copasi software (53).

The model's fitness is approximated by computing the distance z between the mutant and wild-type models, similarly to the minimization of metabolic adjustment (MOMA) approach (54). The distance z represents the deviation from the optimal steady-state in the Euclidean space of the relative values of the four key fluxes $\nu_{9}$ , $\nu_{16}$ , $\nu_{21}$ , and $\nu_{26}$,

[3] $$z={||\frac{\nu -\nu_{0}}{\nu_{0}}||}_{2},$$

with $\nu =\left(\nu_{9},\nu_{16},\nu_{21},\nu_{26}\right)$ the key flux levels in the mutant model, $\nu_{0}$ the wild-type key flux levels, and where the division by $\nu_{0}$ is element-wise. Importantly, this definition assumes that the wild-type model represents a fitness maximum resulting from past evolutionary optimization of erythrocyte metabolism. Note that such an evolutionary optimization may reflect trade-offs between the maximization of key fluxes, and the minimization of enzymatic production costs and metabolite levels due to molecular crowding, osmotic pressure and other broad cellular costs (11, 12). In this model, any mutation in kinetic parameters is deleterious, as it increases the distance z . Moreover, we assume that in the vicinity of the wild-type model, the different kinetic parameters of the same enzyme can be mutated independently without strongly violating thermodynamic constraints, if mutation sizes are small enough (see parameter values in *SI Appendix*, Table S12).

#### Evolutionary simulations using a MCMC approach.

To simulate evolution, we implemented a MCMC modeling algorithm (30). This approach is assumed to be efficient under the weak mutation-strong selection regime (55). During the simulations, no mutation can improve wild-type fitness (i.e., there is no adaptive evolution). Deleterious mutations are deterministically removed by stabilizing selection below a predefined fitness threshold (see below) and neutral mutations can fix by genetic drift with a probability of 1/N_e_, where N_e_ is the effective population size. For computational simplicity, we rescaled the simulation timescale by N_e_, and hence all arising neutral mutations are allowed to fix with a probability of 1 (see below).

As illustrated in *SI Appendix*, Fig. S9, starting from the wild-type model (*SI Appendix*, Fig. S9*A*) and at each iteration t of the MCMC algorithm:

1) One kinetic parameter p is selected at random and mutated (Eq. **2**; *SI Appendix*, Fig. S9*B*),
2) The steady-state of the mutant model is computed (*SI Appendix*, Fig. S9*C*). If the mutant does not reach a steady-state, the iteration t is recalculated (i.e., the mutation is discarded).
3) The distance z between the mutant and the wild-type models is computed (Eq. **3**). Stabilizing selection is simulated by applying a selection threshold ω to the distance z . If z<ω the mutation is accepted. Else, the mutation is discarded (*SI Appendix*, Fig. S9*D*). Thus, no mutation can improve the nonmutated model fitness.
4) A new iteration t+1 is computed (*SI Appendix*, Fig. S9*E*).

We ran 10 repetitions of T=10,000 iterations in two different simulation experiments: i) Genetic drift simulations, where all the mutations are accepted ( ω=+∞ ), and (ii) Stabilizing selection simulations, where a selection threshold $\omega =1\times 10^{-4}$ is applied on the distance z between mutated and nonmutated models, defining a range of selectively neutral mutations that are allowed to fix. For all the simulations, the mutation size was $\sigma_{\mathrm{mut}}=1\times 10^{-2}$ . Simulation parameters are described in *SI Appendix*, Table S12.

The numerical framework (as a Python package), simulation results, and scripts for additional analyses are publicly available on GitHub (https://github.com/pappb/Liska-et-al-Principles-of-metabolome-conservation).

#### In silico MCS.

At the end of the evolutionary simulation, the evolutionary rate of each metabolite level is calculated based on a BM estimation model (18),

[4] $$\mathrm{ER}\left(\left[X_{i}\right]\right)=\frac{\mathrm{var}\left({\left[X_{i}\right]}_{t}/{\left[X_{i}\right]}_{0}\right)}{T},$$

with $\mathrm{ER}\left(\left[X_{i}\right]\right)$ the evolution rate of the level $\left[X_{i}\right]$ of metabolite $X_{i}$ , ${\left[X_{i}\right]}_{t}$ being its level at iteration t , ${\left[X_{i}\right]}_{0}$ the level of the wild-type model, and T the total number of iterations of the simulation. The conservation score of each metabolite is then calculated by taking the inverse of the evolution rate.

#### Calculating the fitness coupling of metabolites.

We defined a fitness coupling measure for each metabolite by introducing many independent random single mutations into the kinetic parameters of the model and by calculating the Spearman correlation coefficient between the relative change of metabolite levels and the relative change of the four key metabolic fluxes. To this aim, we performed N=10,000 independent single mutations of the wild-type model, by selecting a single kinetic parameter at random uniformly among reactions, and mutating it in a log_10_-normal distribution of size ${\sigma_{\mathrm{mut}}}^{2}=1\times 10^{-2}$ . We measured each time the relative change of metabolite levels and key fluxes in response to mutations. We then used this result to compute pairwise correlations between fluxes and metabolites. Specifically, for each steady-state flux $\nu_{j}$ and each steady-state metabolite level $\left[X_{i}\right]$ across all mutations, the Spearman correlation was computed between the absolute value of relative changes, compared to the wild-type model.

The fitness coupling of a given metabolite $X_{i}$ was then evaluated by computing the mean correlation between the metabolite and the four key fluxes $\nu_{9}$ , $\nu_{16}$ , $\nu_{21}$ , and $\nu_{26}$:

[5] $$\mathrm{coupling}\left(X_{i}\right)=\frac{1}{4}\times \sum_{j\in \left\{9,16,21,26\right\}}\mathrm{cor}\left(\left|\frac{\left(\nu_{j}-\nu_{j,0}\right)}{\nu_{j,0}}\right|,\left|\frac{\left(\left[X_{i}\right]-{\left[X_{i}\right]}_{0}\right)}{{\left[X_{i}\right]}_{0}}\right|\right),$$

where $\nu_{j,0}$ is the wild-type value of $\nu_{j}$ , and ${\left[X_{i}\right]}_{0}$ is the wild-type value of $X_{i}$.

#### Exploration of random combinations of key metabolic fluxes.

We also used the methodology described above to compute the coupling of metabolites (Eq. **5**) to 10,000 combinations of key fluxes drawn at random (from random 1-uplets to 4-uplets). For each random combination, the fitness couplings of metabolites were computed, as well as the Spearman correlation between metabolite abundances and their fitness coupling. For 100 random combinations of key fluxes, we also computed one stabilizing selection simulation ( $\omega =1\times 10^{-4}$ , $\sigma_{\mathrm{mut}}=1\times 10^{-2}$ , and T=10,000 ) per combination, in order to compute the Spearman correlation between metabolite abundances and their conservation scores.

#### Calculating in silico reaction essentiality.

For each of the 38 reactions of the model, we reduced the flux level to a small fraction of the wild-type level (0.001% for the erythrocyte model from ref. 29), computed the new steady-state and evaluated the deviation of the four key fluxes relative to their wild-type level. This measure quantifies the essentiality of each reaction regarding deviation from optimal key flux levels and hence fitness. We considered a reaction as essential if at least one key metabolic flux is dropped to zero upon its inhibition. Metabolites that are substrates or products of at least one essential reaction are classified as “essential metabolites.” We were able to determinate the essentiality of 32 metabolites. It was not possible to calculate it for three metabolites because of numerical stability issues.

#### Removal of low-varying metabolites.

From all modeling analyses, we excluded five metabolites whose variability was either zero or underestimated in evolutionary simulations: NAD (nicotinamide adenine dinucleotide), P1NADPH (reduced nicotinamide adenine dinucleotide phosphate), glutathione, pyruvate, and lactate. These metabolites are insensitive to mutations, as the variabilities of NAD, P1NADPH, and glutathione are zero in some genetic drift simulations, while pyruvate and lactate are directly dependent on constant input/output metabolites through transport reactions.

### Metabolites Associated with Human Diseases.

Metabolites associated with IEM were compiled as follows. We included 24 IEM diseases from the US Health Resources and Services Administration’s core recommended uniform newborn screening panel (https://www.hrsa.gov/advisory-committees/heritable-disorders/rusp). We identified disease-associated metabolites by manual curation from the relevant literature, as well as the Online Mendelian Inheritance in Man database (https://www.omim.org/) and the Orphanet database of rare diseases (https://www.orpha.net). Any metabolite whose level is known to be affected by the disease-causing mutation or is known to show an altered level on diagnostic panels was classified as being associated with the IEM disease.

We tested the difference in conservation scores between IEM associated and nonassociated metabolites in all four measured organs using ANOVA. To test whether the results are not biased by amino acids, which are prevalent among IEM-associated metabolites, we repeated the test after excluding all metabolites that are classified as “amino acids, peptides, and analogues” according to ref. 7. To ensure that the high conservation of IEM-associated metabolites is not driven by single specific metabolite pathways, we identified five metabolic pathways in KEGG that include three or more IEM associated metabolites: “alanine, aspartate, and glutamate metabolism,” “arginine biosynthesis,” “phenylalanine metabolism,” “valine, leucine, and isoleucine biosynthesis,” and “valine, leucine, and isoleucine degradation.” We then repeated the ANOVA test five times, excluding each one of the above pathways in turn.

For the expanded disease association analysis, we collected chemical biomarkers from the MarkerDB database (32). The database includes the known chemical biomarkers of a total of 407 human diseases, all of which belong to at least one of 20 broad disease conditions present in MarkerDB. Note that all conditions that are listed in the categories “others” (such as pregnancy) and “exposure” (such as smoking) only were omitted from further analysis, as most of these are not strictly disease conditions. In total, 106 metabolites in our dataset were associated with at least one broad disease condition.

To probe the associations between conservation score and involvement of metabolites in broad disease conditions, we focused on 11 broad disease conditions, each of which is associated with at least 10 metabolites in our dataset. The 11 broad conditions include cancers, cardiovascular system disorders, digestive system disorders, endocrine disorders, germ line disorders, hematological and lymphatic disorders, immune disorders, mental and behavioral disorders, metabolic disorders, nervous system disorders, and urinary system disorders. Because the same metabolite might be involved in multiple broad disease conditions, we used a multivariate approach to determine which disease conditions are significantly associated with MCS while controlling for the effects of other disease conditions. First, we determined which broad disease conditions’ biomarkers are significantly more conserved than nonbiomarker metabolites using univariate two-sided Wilcoxon rank-sum tests (*P* < 0.05 in at least three out of the four organs). Then, we determined which of the remaining disease conditions show significant independent associations with conservation score using a multivariate linear regression model.

To estimate the extent to which metabolites associated with metabolic disorders in MarkerDB can be predicted based on MCS, we first calculated an aggregate conservation score for each metabolite that represents its level of conservation across the four organs. This was achieved by first normalizing the conservation scores in each organ (see *Between-Organ Differences in MCS*), and then taking the median value across the four organs as an aggregate MCS. We then built a classification model using logistic regression that predicts association with metabolic disorders using only the aggregate MCSs. We then evaluated the prediction accuracy of the classifier by a receiver operating characteristics (ROC) curve analysis and by calculating AUC using the R package “ROCR” (56). To test the relationship between a metabolite’s conservation score and the number of associated diseases, we used Spearman’s correlation. This analysis included all specific metabolite–disease associations from MarkerDB, not just those involving the 11 broad disease condition categories.

### Metabolome Conservation in the *Drosophila* Genus.

Metabolomics data of 92 nonlipid metabolites measured in 11 *Drosophila* species (*D. ananassae, D. yakuba, D. erecta, D. melanogaster, D. simulans, D. sechellia, D. pseudoobscura, D. persimilis, D. willistoni, D. virilis,* and *D. mojavensis*) and the phylogenetic tree describing the evolutionary relationship between the species were obtained from ref. 35. Conservation scores were computed as described above (see “*Calculating MCSs*”) (35).

Absolute metabolite concentrations of 35 nonlipid metabolites, as quantified by NMR metabolomics, were obtained from ref. 36. We used the metabolite concentrations measured in whole *D. melanogaster* larvae samples, in order to best match the samples used in ref. 35. Of these 35 metabolites, 24 overlapped between the datasets.

## Supplementary Material

Appendix 01 (PDF)

Dataset S01 (XLSX)

## Data Availability

All data associated with this study are available in the supporting information. Data and code associated with the systems modeling work is available on GitHub (https://github.com/pappb/Liska-et-al-Principles-of-metabolome-conservation) (57).
