# Supplementary material for: Principles of metabolome conservation in animals
Source: Proc Natl Acad Sci U S A. 2023 Aug 21;120(35):e2302147120. doi: 10.1073/pnas.2302147120 (PMC10468614; doi:10.1073/pnas.2302147120)
Supplement: Supplementary file 1 — Appendix 01 (PDF) [file pnas.2302147120.sapp.pdf]

Supporting Information for
Principles of metabolome conservation in animals

Orsolya Liska, Gábor Boross, Charles Rocabert, Balázs Szappanos, Roland Tengölics, Balázs Papp

Balázs Papp
Email: pappb@brc.hu

This PDF file includes:

- Figures S1 to S15
- Tables S1 to S12
- Appendix S1
- SI References

Other supporting materials for this manuscript include the following:

- Dataset S1

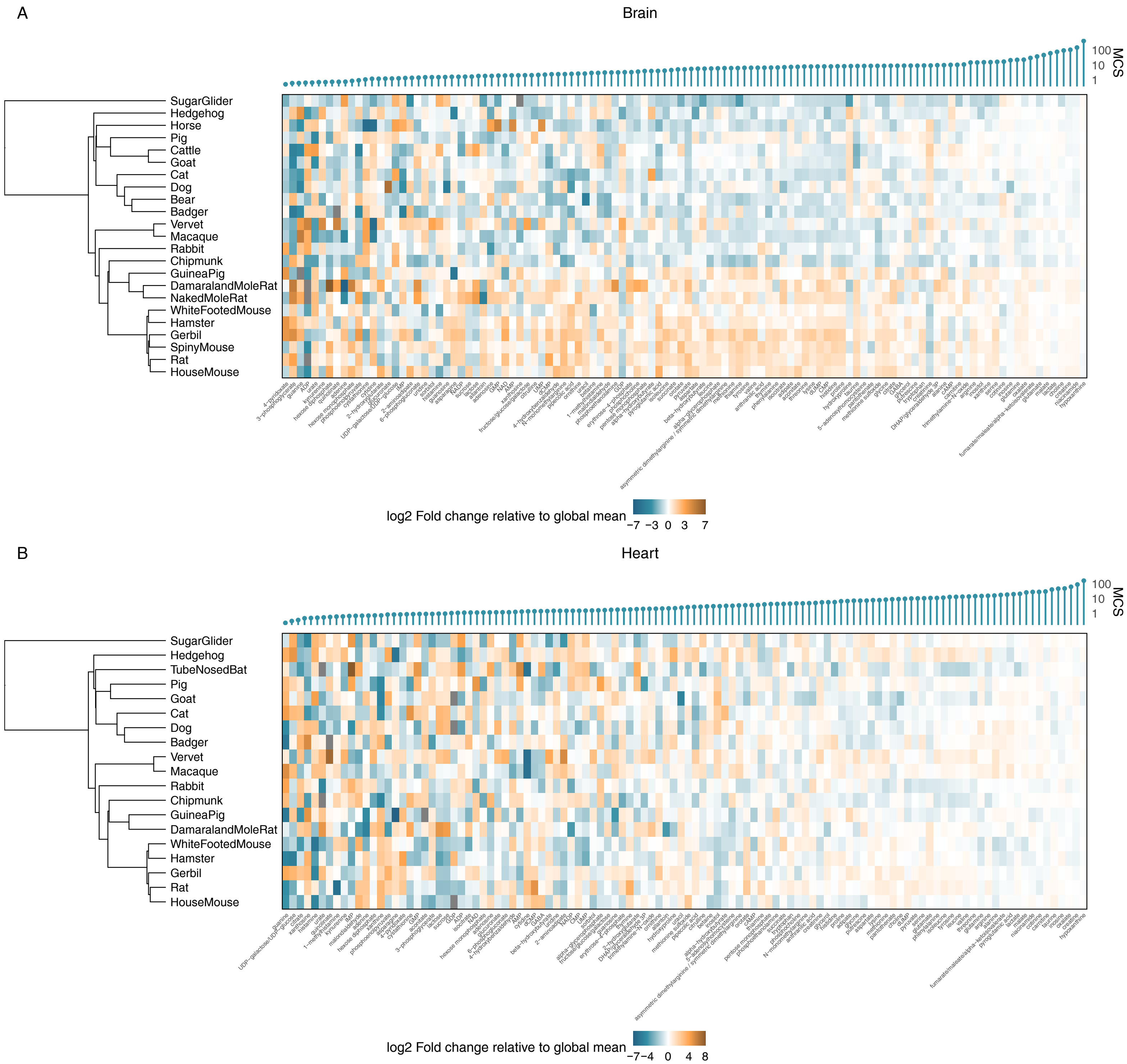
A
Brain
MCS
100
10
1
SugarGlider
Hedgehog
Horse
Pig
Cattle
Goat
Cat
Dog
Bear
Badger
Vervet
Macaque
Rabbit
Chipmunk
GuineaPig
DamaralandMoleRat
NakedMoleRat
WhiteFootedMouse
Hamster
Gerbil
SpinyMouse
Rat
HouseMouse
log2 Fold change relative to global mean
−7 −3 0 3 7
B
Heart
MCS
100
10
1
SugarGlider
Hedgehog
TubeNosedBat
Pig
Goat
Cat
Dog
Badger
Vervet
Macaque
Rabbit
Chipmunk
GuineaPig
DamaralandMoleRat
WhiteFootedMouse
Hamster
Gerbil
Rat
HouseMouse
log2 Fold change relative to global mean
−7−4 0 4 8

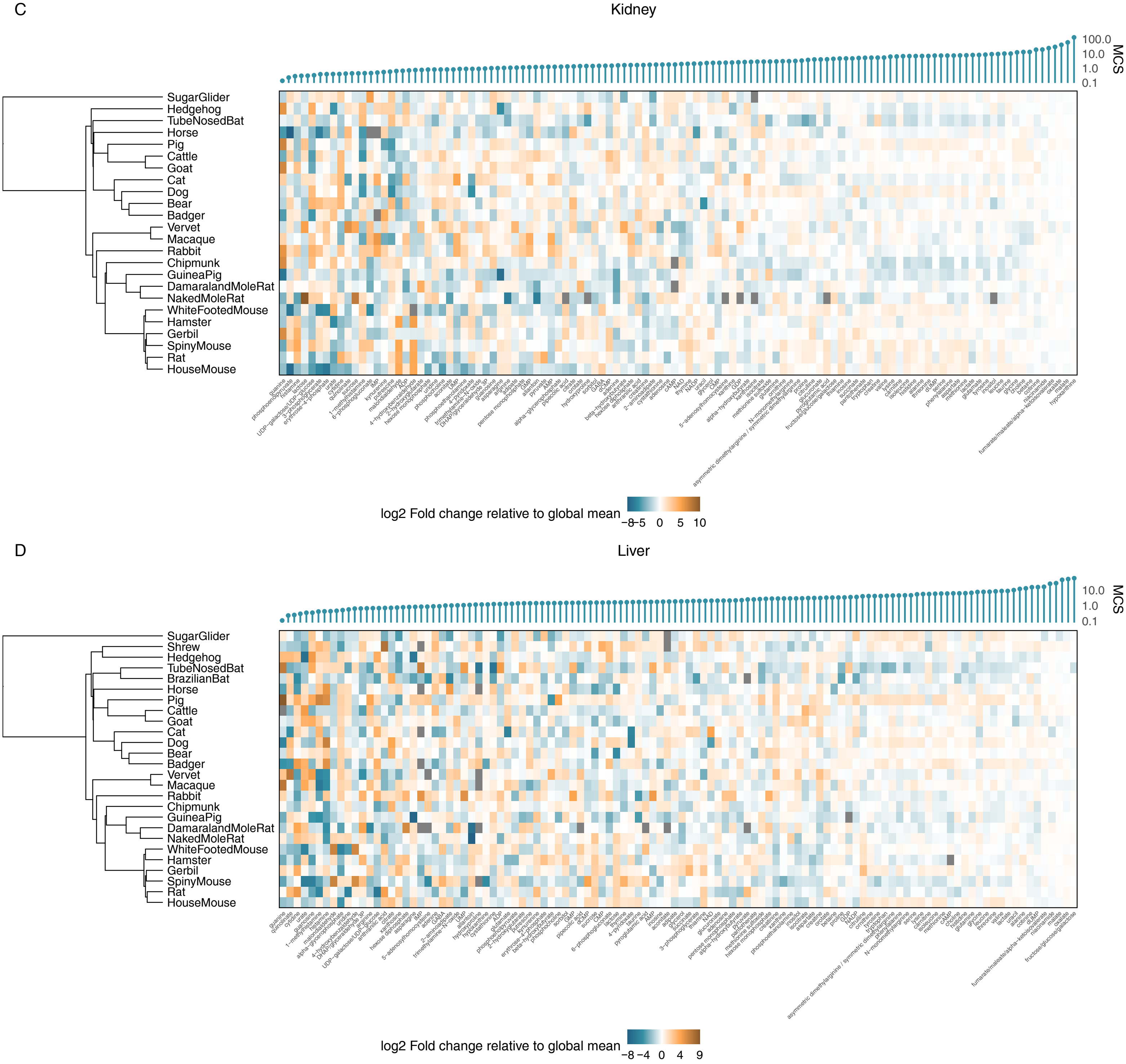

Figure S1: Metabolites differ extensively in their between-species metabolite level variability.

Plot shows the phylogeny of the mammalian species used in the study and heatmaps illustrating how metabolite levels vary among species in brain (A), heart (B), kidney (C) and liver (D), sorted by metabolite conservation scores (MCS). Colors of the heatmaps represent, in each organ, the log2 fold-change between the level of each metabolite measured in the given species, and the average metabolite level of all species (number of species= 23, 19, 24 and 26 for brain, heart, kidney and liver, respectively). Grey color indicates missing data (i.e. metabolites that were not measured in the given organ and species). Number of metabolites are 121, 126, 126 and 132 for brain, heart, kidney and liver, respectively. Lollipop charts above the heatmaps show the MCS of each metabolite in each organ. Metabolites with lower between-species variance (lighter colors) tend to be more evolutionary conserved.

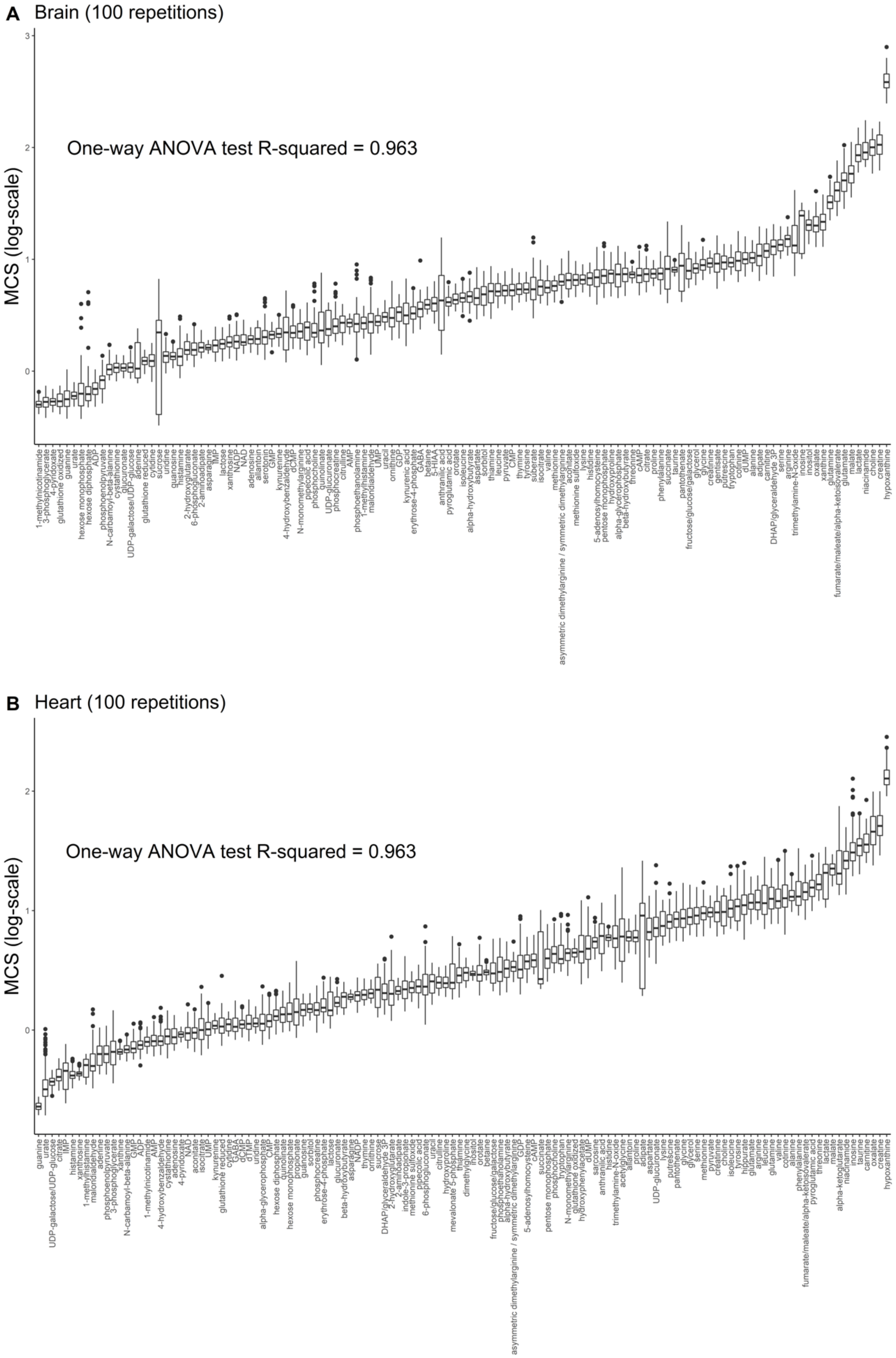

A
Brain (100 repetitions)
One-way ANOVA test R-squared = 0.963
MCS (log-scale)
B
Heart (100 repetitions)
One-way ANOVA test R-squared = 0.963
MCS (log-scale)

C Kidney (100 repetitions)

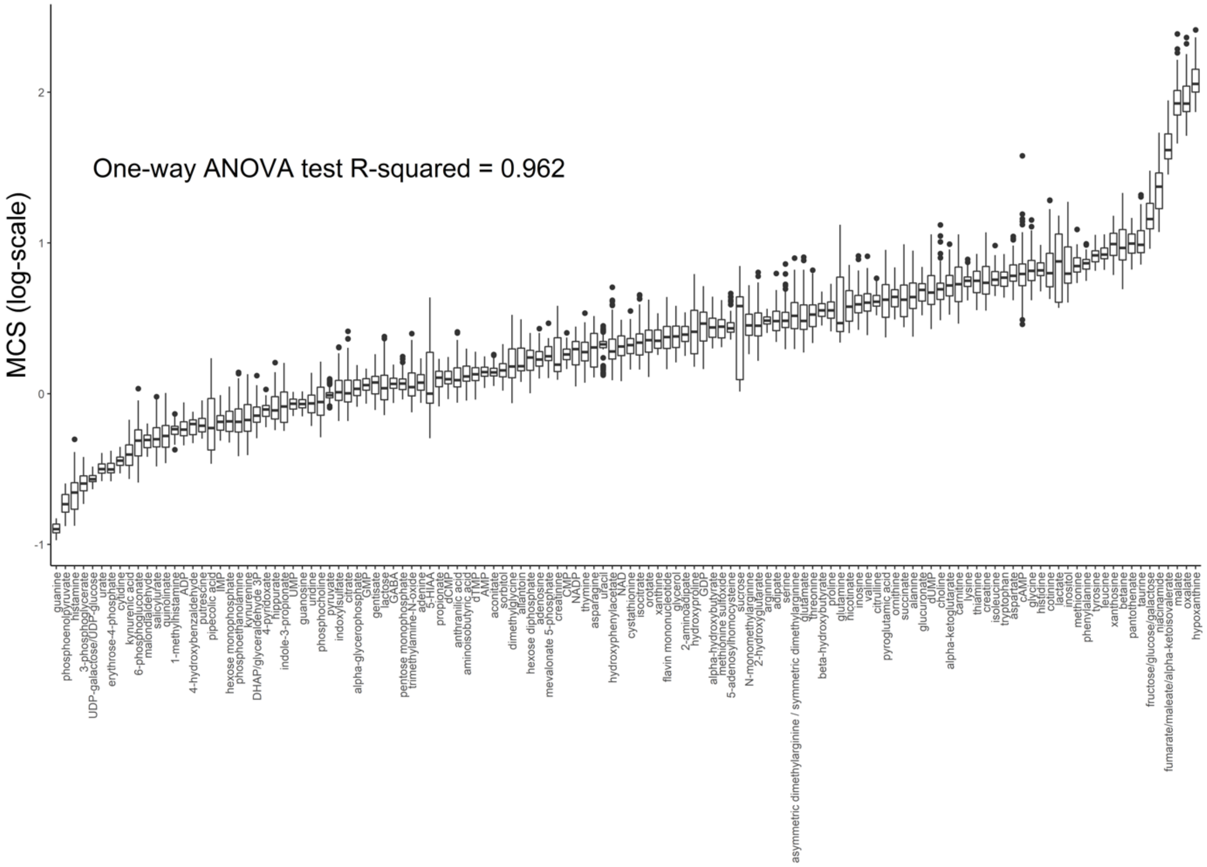

D Liver (100 repetitions)

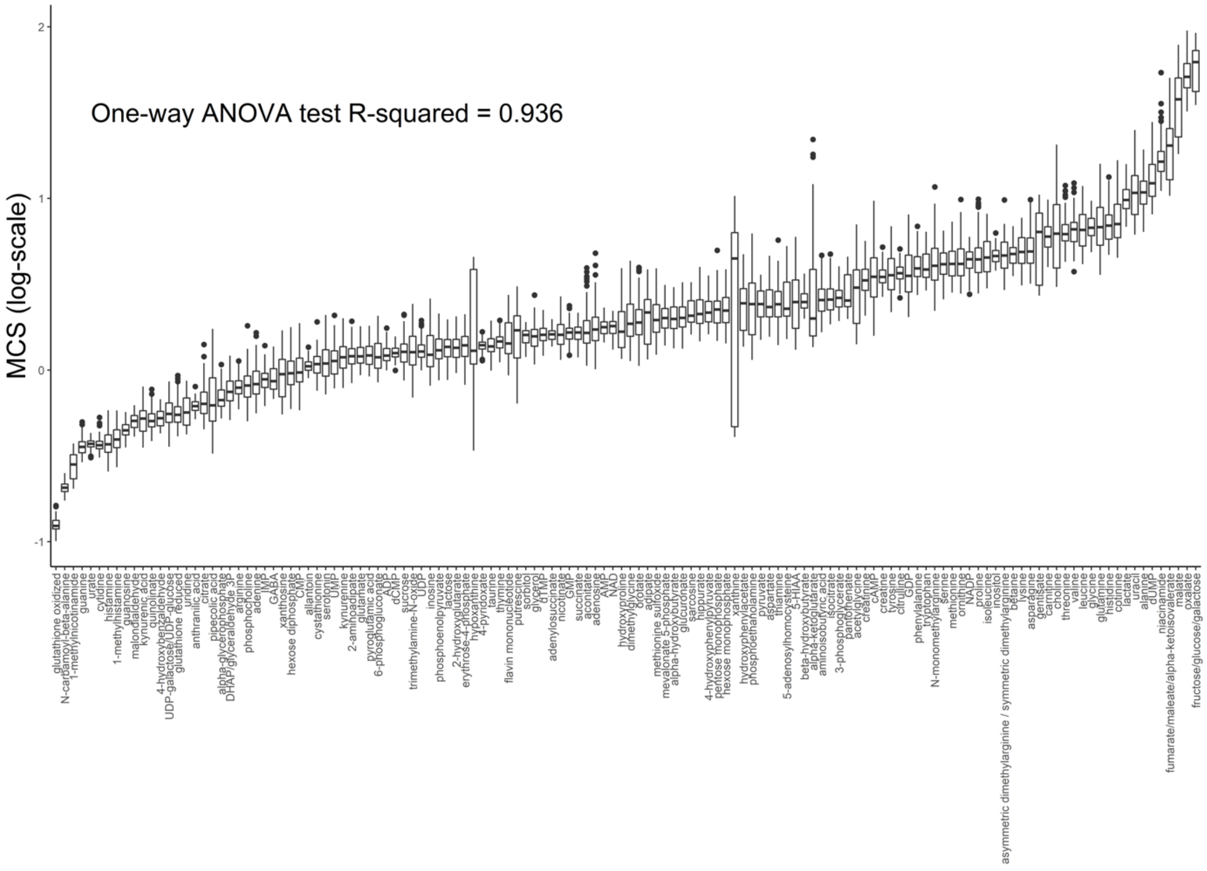

Figure S2: Variation of MCSs among metabolites versus biological replicate measurements. For each organ, we computed a one-way ANOVA to estimate the percentage of total variance of metabolite conservation scores (MCS) explained by between-metabolite differences versus differences between biological replicate measurements based on resampling of replicates (see Methods). Box-plots show the distribution of metabolite conservation score estimates calculated from 100 bootstrap samples of biological replicate measurements (see Methods). For each organ, metabolites are ranked by the mean value of their conservation scores. The R-squared values are shown, and all the p-values associated to ANOVA tests are $< 2.2 \times 10^{-16}$. **A** Brain ($R^2 = 0.963$). **B.** Heart ($R^2 = 0.963$). **C.** Kidney ($R^2 = 0.962$). **D.** Liver ($R^2 = 0.936$).

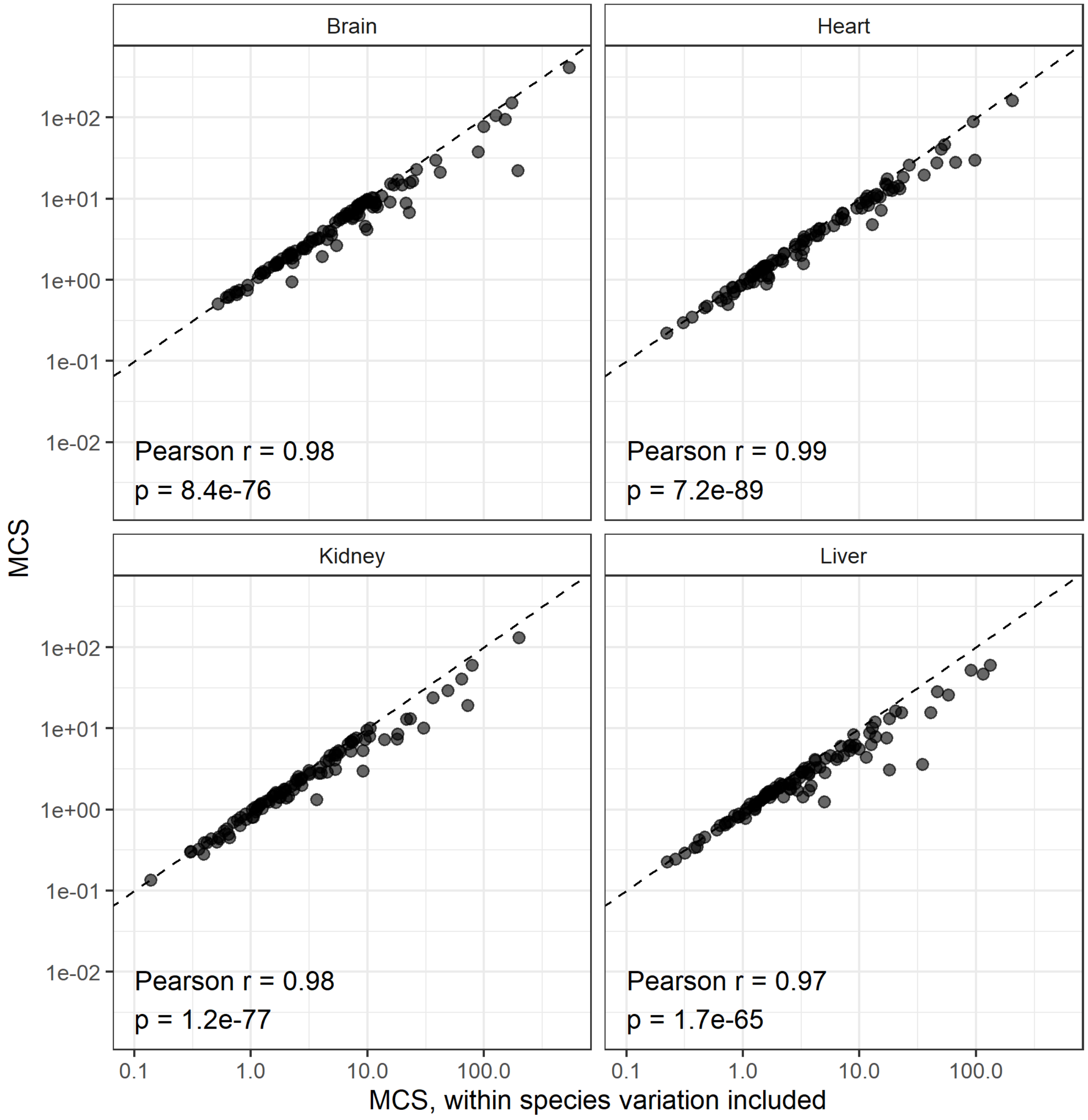

Figure S3: Conservation score estimates show remarkably strong correlation with those calculated while incorporating within-species measurement noise. While metabolite conservation scores that account for within-species variation as well are generally lower, they show remarkably strong correlation with the scores calculated based on the metabolite measurements only (N_{Brain} = 113, N_{Heart} = 105, N_{Kidney} = 102, N_{Liver} = 108).

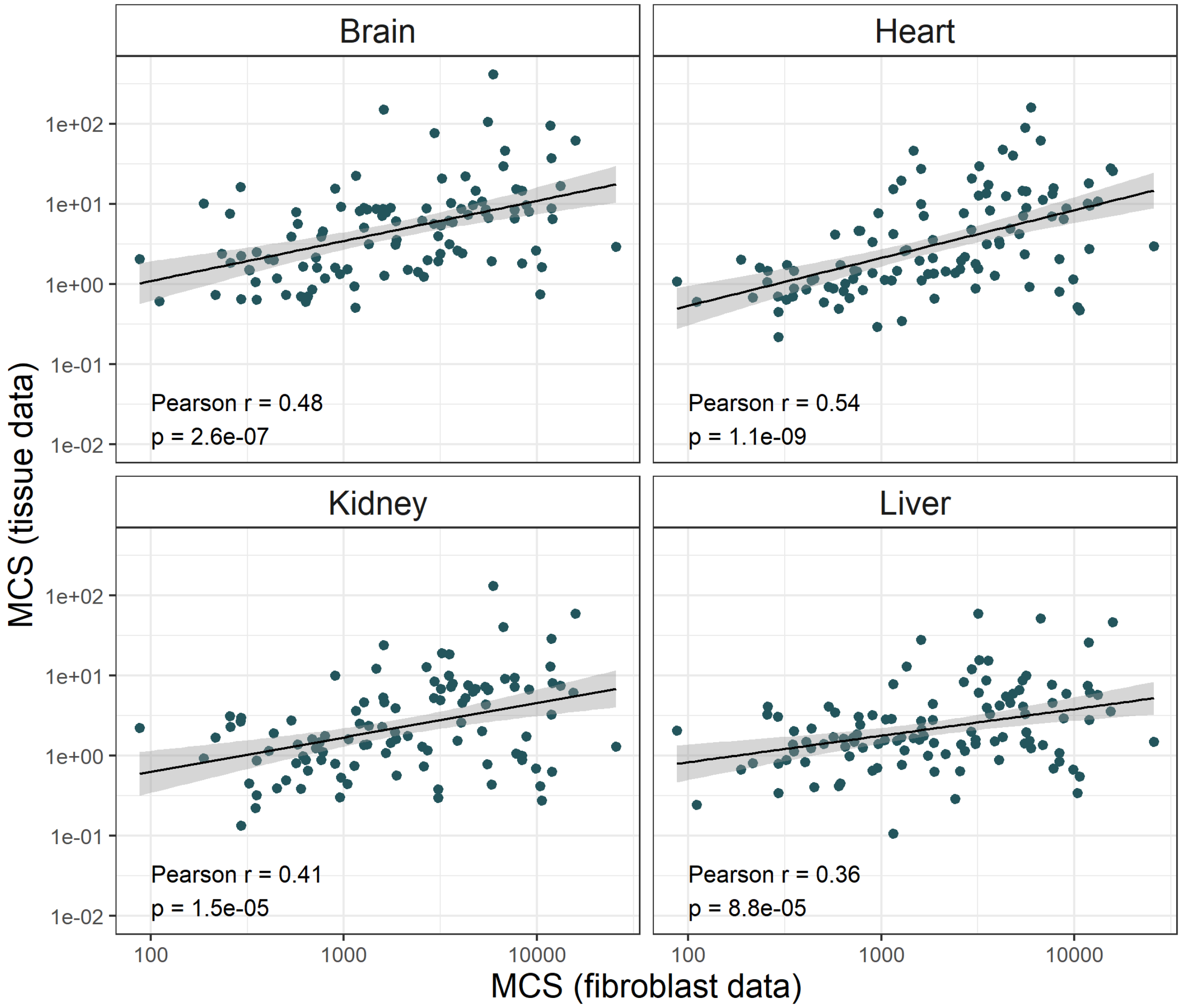

Figure S4: Metabolite conservation scores inferred from *in vivo* mammalian organ samples correlate with those based on metabolome measurements in cell cultures. Scatterplots show the correlation between the conservation scores of metabolites in mammalian organ samples (y-axis) and those measured in fibroblast cell cultures (x-axis) (N_{Brain} = 105, N_{Heart} = 110, N_{Kidney} = 106, N_{Liver} = 111). Note that the same metabolomic pipeline was used for both datasets. The lines represent linear regressions, with their 95% confidence intervals shaded in grey. All four organs showed significant positive correlation with fibroblast derived conservation scores.

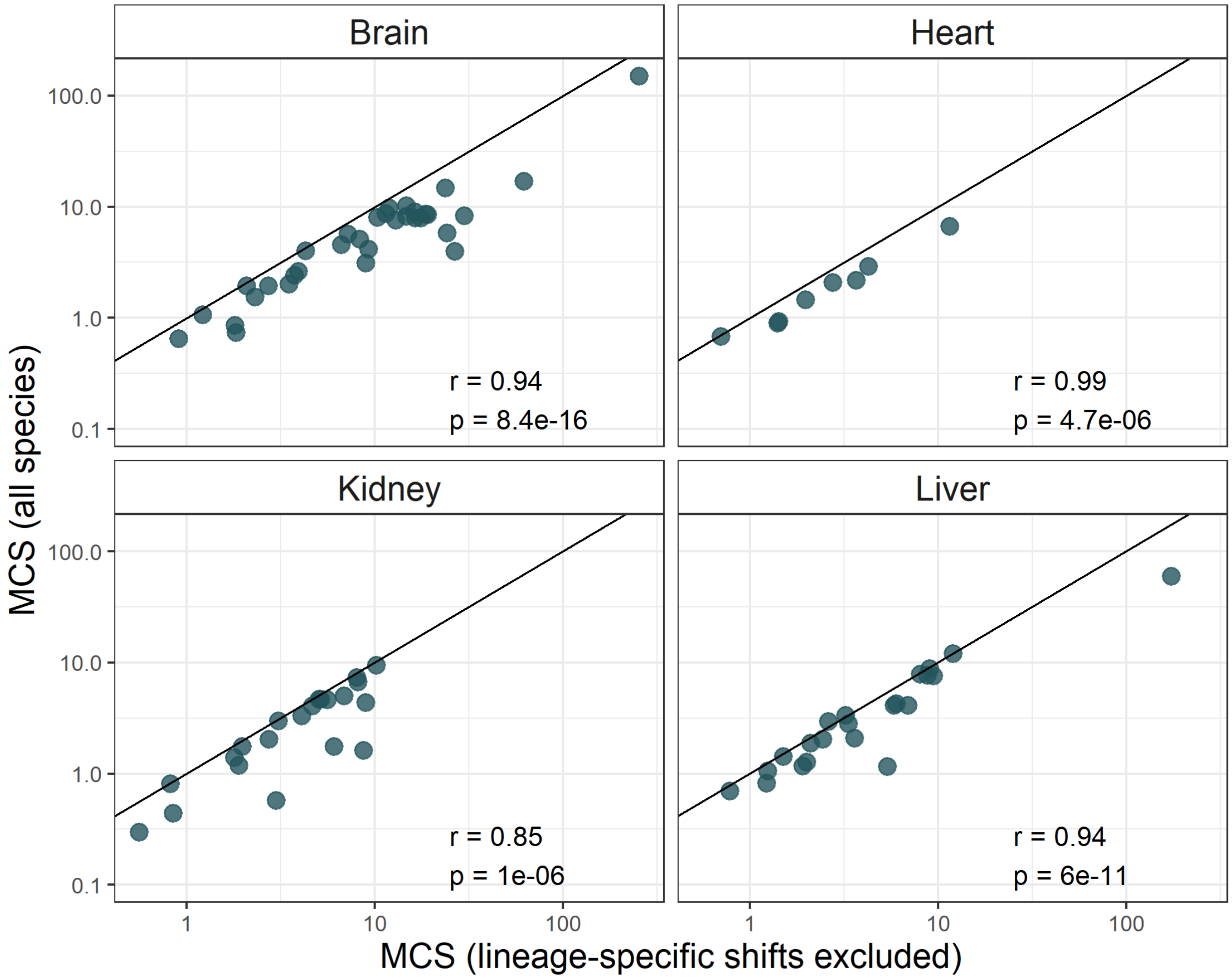

Figure S5: **Impact of lineage-specific evolutionary shifts in metabolite levels on MCS inferences.** For each organ, we focused on metabolites that show evidence for lineage-specific shifts in their levels according to Ma et al. (1). A total of 33, 8, 21 and 22 metabolites show lineage-specific changes in their levels in brain, heart, kidney and liver, respectively. For these metabolites, we calculated metabolite conservation scores (MCS) after excluding those species where lineage-specific changes occurred (1). In all four organs, these MCSs show strong positive correlations with those calculated based on all measured species based on Pearson correlations of log-scaled MCSs. The line in the figures depicts the identity function (slope of 1).

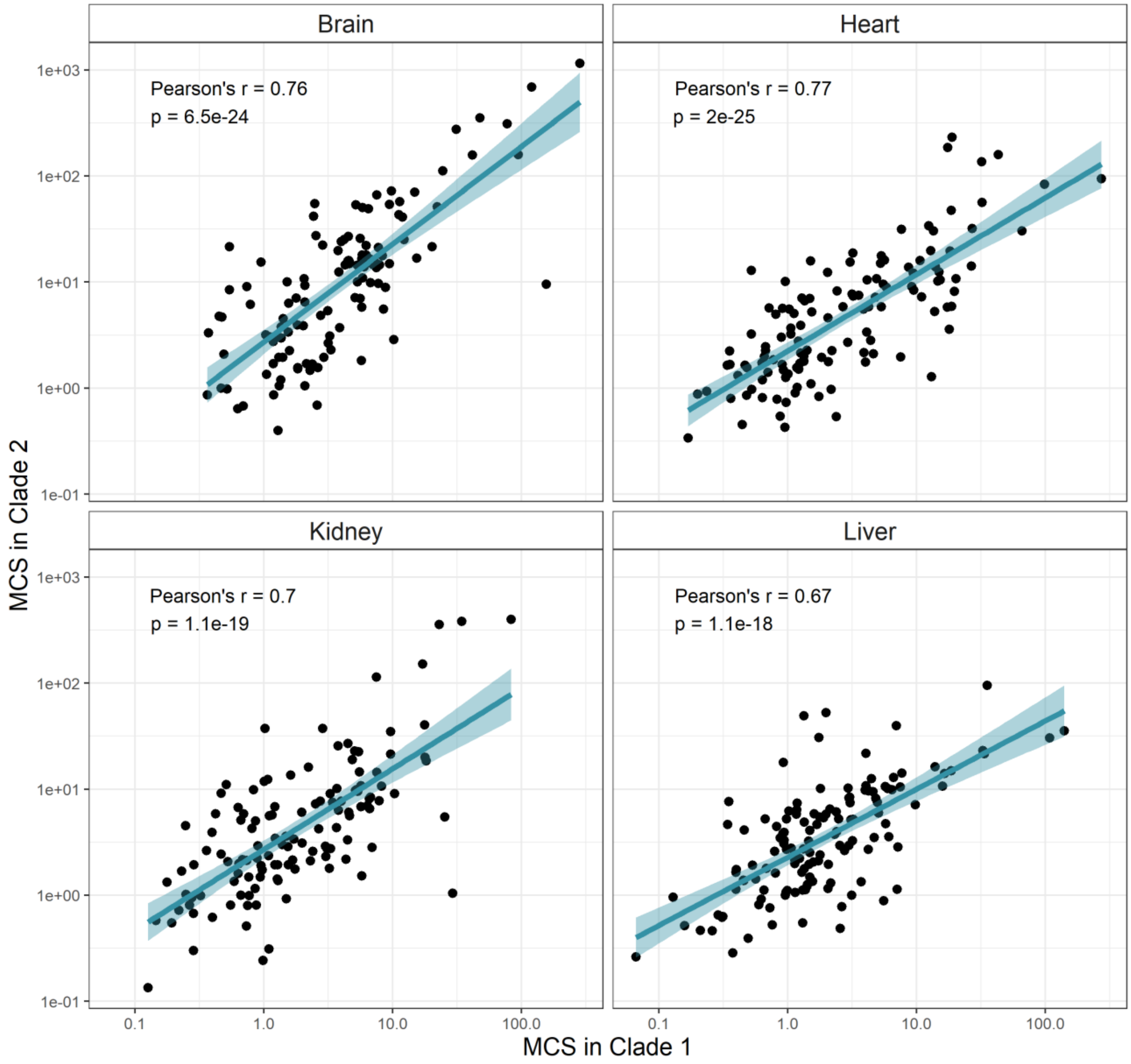

Figure S6: Metabolite conservation scores calculated on two independent clades of the tree are well correlated in all four organs. Pearson correlations are significant in all four organs (N_{Brain} = 121, N_{Heart} = 125, N_{Kidney} = 126, N_{Liver} = 132). Lines depict the fitted linear regression. See main figure Fig. 1C for the definition of Clade 1 and Clade 2.

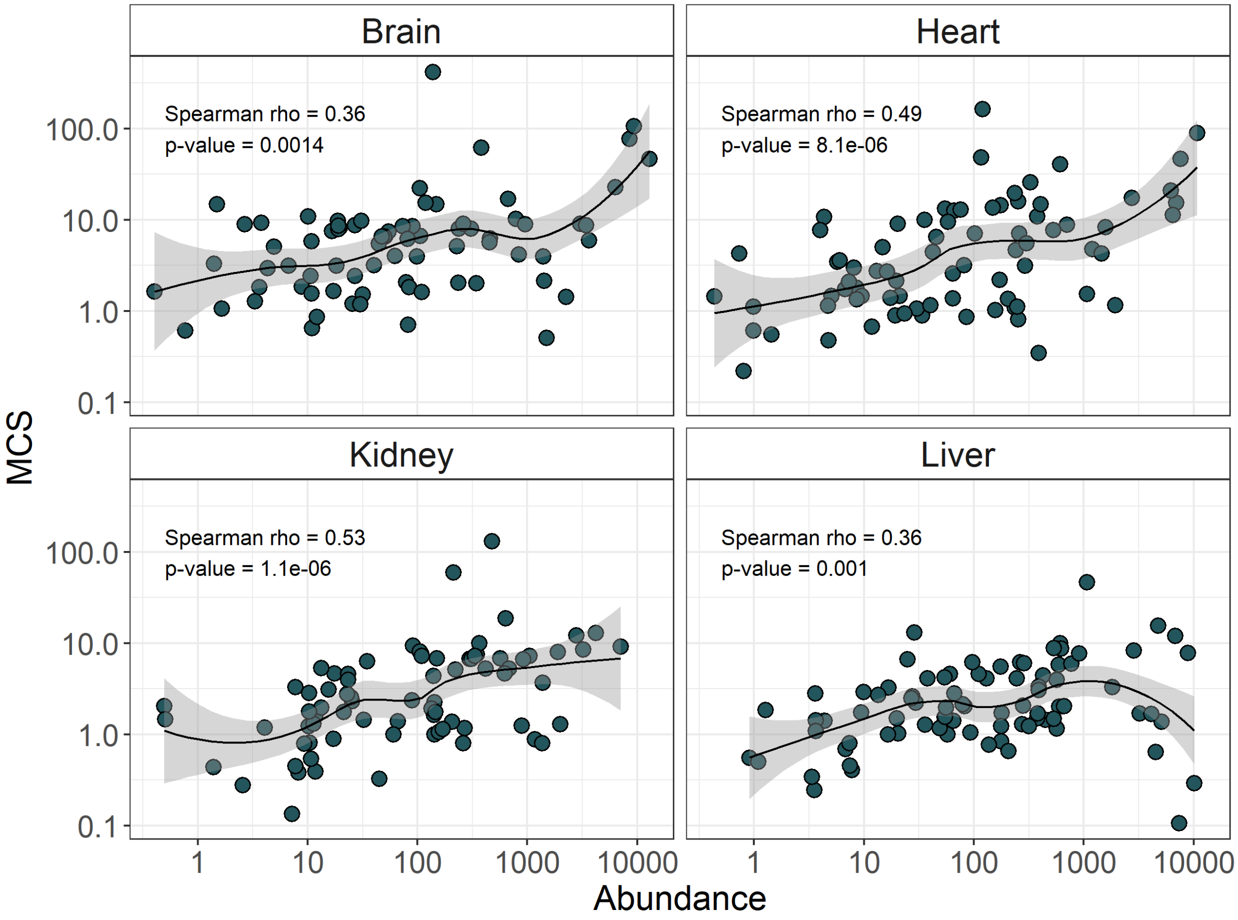

Figure S7: **Highly abundant metabolites tend to be more conserved in their levels in all organs.** Scatter plots show relationships between metabolite conservation scores of and absolute metabolite abundances (both log_{10}-scaled, N_{Brain} = 77, N_{Heart} = 78 N_{Kidney} = 76, N_{Liver} = 83). We used abundance data from an independent study quantifying absolute metabolite concentrations in mice from matched organs (2). Lines indicate smooth curves fitted using LOESS regression, with their 95% confidence intervals shaded in grey.

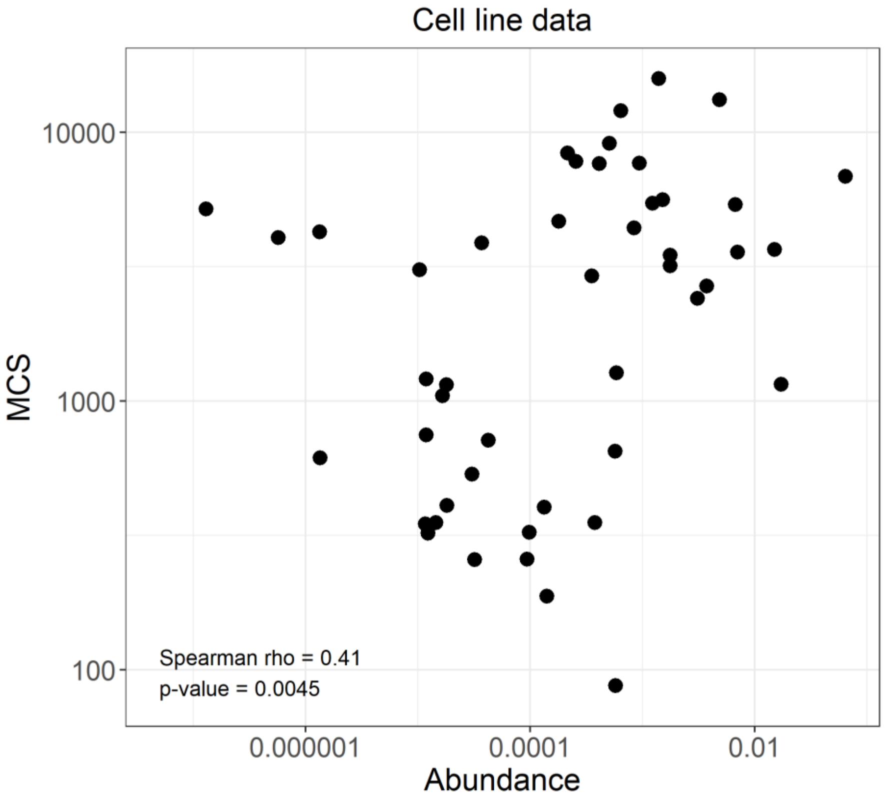

Figure S8: **Cell culture-based metabolome data confirms the high MCS of abundant metabolites**. Metabolite conservation scores were calculated from metabolome measurements of primary skin fibroblasts of 16 mammalian species (relative concentrations) (3). As absolute metabolite concentration was not available for fibroblast cultures, we used absolute concentration data on a mouse cell line derived from baby mouse kidney cells (iBMK) (4). We found a significant positive correlation between absolute metabolite abundance and conservation scores (Spearman's rho = 0.41, p-value = 0.0045, N = 47).

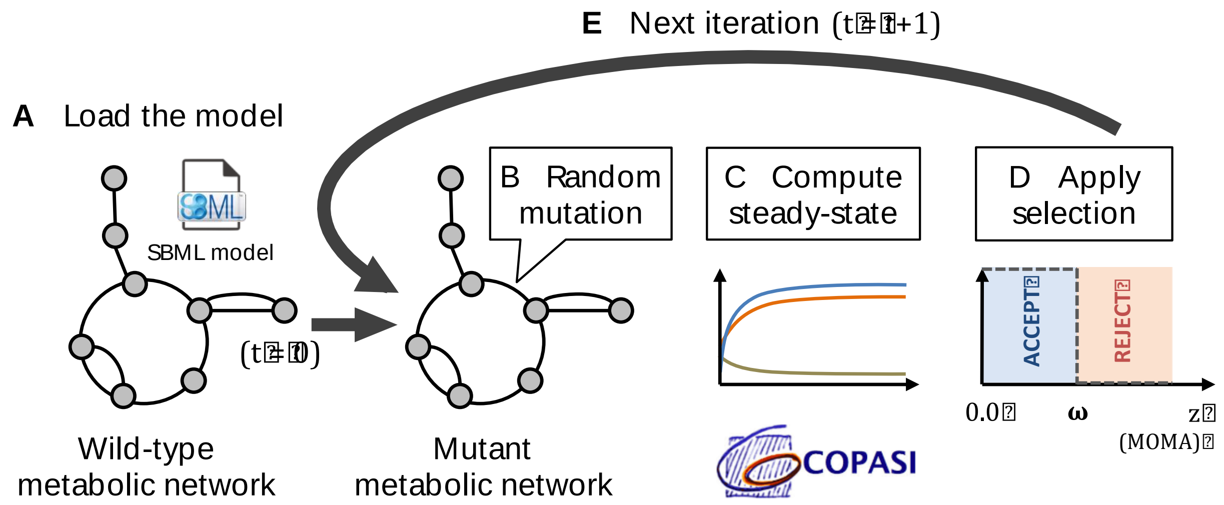

Figure S9: Overview of the algorithm to simulate the evolution of metabolite levels. A. The model of interest is loaded as a wild-type from an SBML file (5). Kinetic equations, kinetic parameter values and initial metabolite concentrations must be specified. **B.** At each iteration t, a single kinetic parameter is selected at random and mutated through a $\log_{10}$-normal distribution of standard deviation σ_{mut}. **C.** The new steady-state is computed using the Copasi software (6), and the deviation z of key metabolic fluxes in the mutant relative to their wild-type is computed (see Methods). **D.** If z is under a given selection threshold ω ($z < \omega$) the mutation is accepted. Otherwise, the mutation is rejected (mutants must reach a stable steady-state to accept the iteration). **E.** A new iteration $t + 1$ is computed.

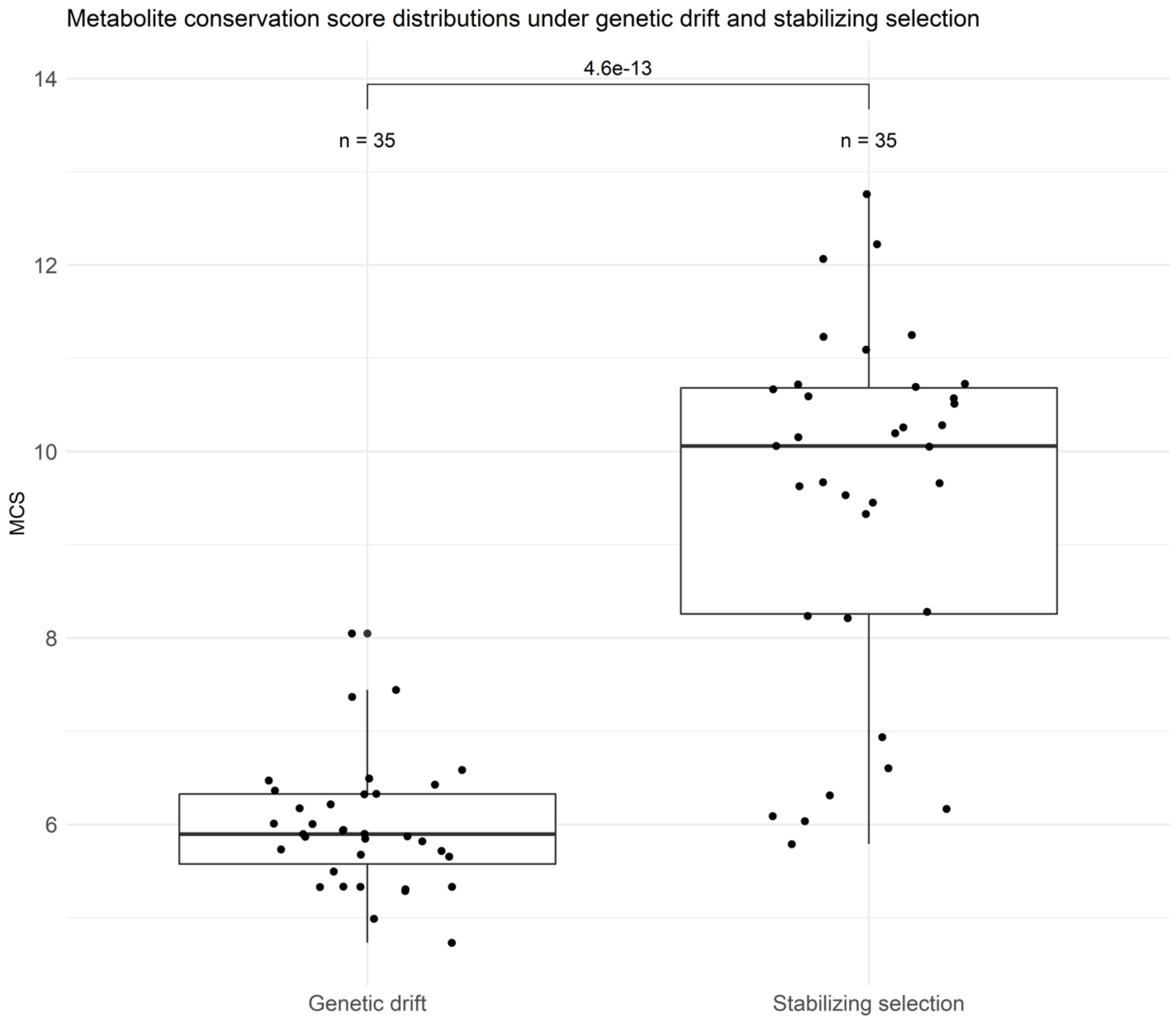

Figure S10: Distribution of simulated metabolite conservation scores under genetic drift and stabilizing selection. The distribution of simulated MCSs of all metabolites are shown for both selection regimes (pure genetic drift versus stabilizing selection). The median of metabolite conservation scores differ significantly between the two selection regimes (Welch two-sample t-test with unequal variance, p-value $= 4.57 \times 10^{-13}$). MCSs show larger variance under stabilizing selection as compared to pure genetic drift (F-test, F = 0.127, p $<10^{-7}$).

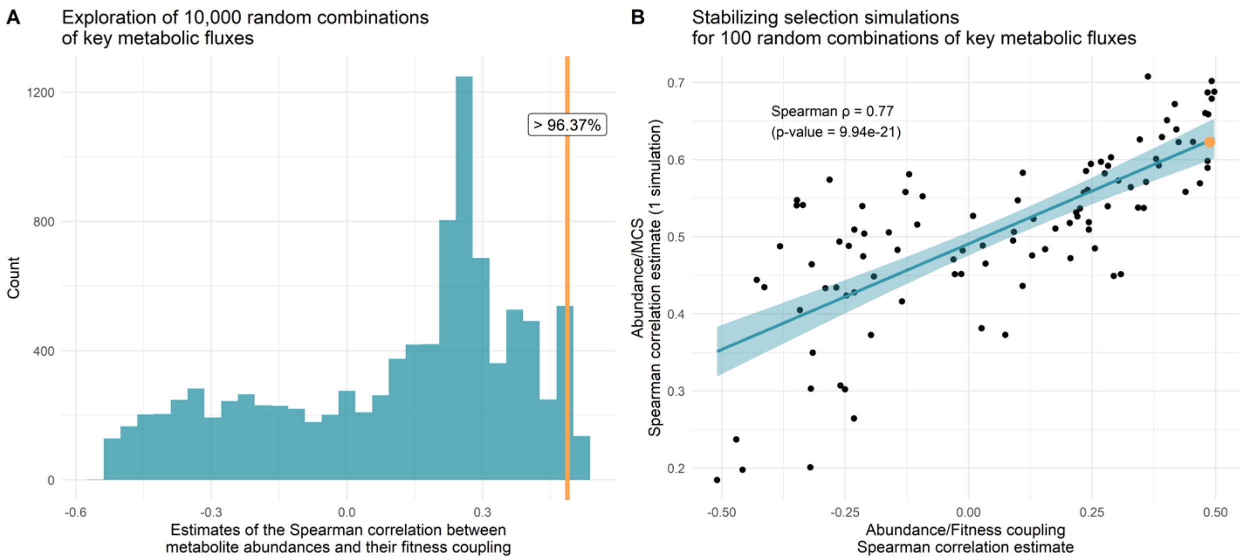

Figure S11: The relationship between metabolite abundance and fitness coupling for randomly defined key fluxes. A. The correlation between metabolite abundance and fitness coupling is especially strong when approximating fitness with the four key fluxes assumed to be important for erythrocyte function compared to randomly defined key fluxes. For the analysis, we calculated the fitness coupling of each metabolite to 10,000 randomly defined combinations of key fluxes (uniformly distributed from 1-uplets to 4-uplets), represented by 10,000 biochemical models. Then, the Spearman correlation is computed between metabolite abundances and their fitness coupling for each of the 10,000 model. The distribution of the 10,000 Spearman estimates is shown. The orange line represents the Spearman correlation coefficient based on the default key fluxes (ν_9, ν_{16}, ν_{21} and ν_{26}; (7)), which is higher than 96.37% of the 10,000 random combinations. **B.** Evolutionary simulations with stabilizing selection were run for 100 metabolic models with randomly defined combinations of key metabolic fluxes (see Methods). At the end of each simulation, the Spearman correlation between metabolite abundance and *in silico* MCS was computed and plotted against the Spearman correlation between metabolite abundance and their fitness coupling calculated in the same model. The two measures show a strong positive correlation (Spearman $\rho = 0.77$, p-value $= 1.76 \times 10^{-20}$). The bold orange dot represents the estimates for the default key fluxes (ν_9, ν_{16}, ν_{21} and ν_{26}) (7). Thus, the correlation between metabolite abundance and *in silico* MCS is strongest in those specific biochemical models where abundant metabolites are strongly coupled to

key fluxes. This results indicates that the high evolutionary conservation score of abundant metabolites is caused by their strong coupling to key fluxes in the model.

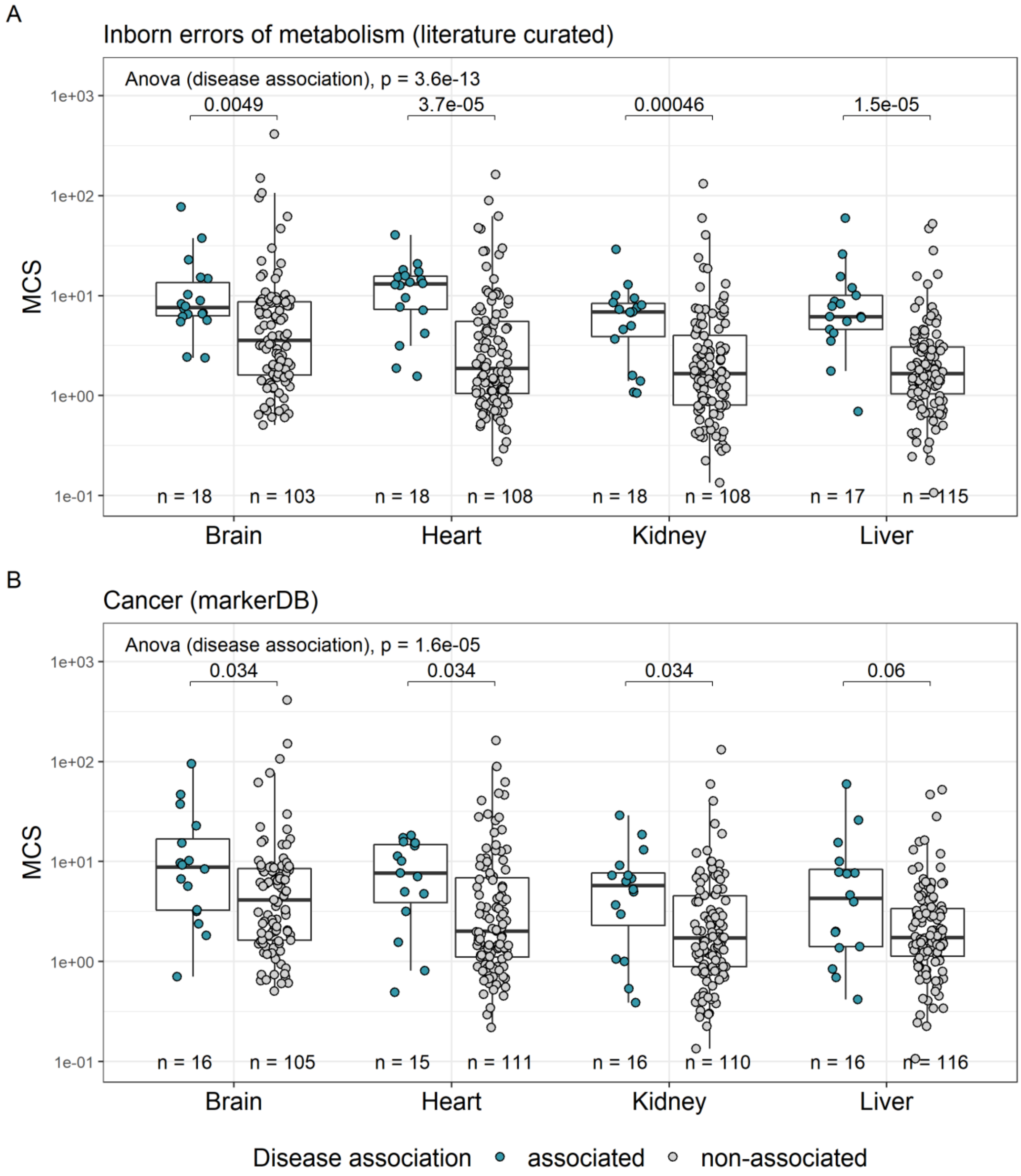
A
Inborn errors of metabolism (literature curated)
Anova (disease association), p = 3.6e-13
0.0049
3.7e-05
0.00046
1.5e-05
MCS
1e+03
1e+02
1e+01
1e+00
1e-01
n = 18
n = 103
n = 18
n = 108
n = 18
n = 108
n = 17
n = 115
Brain
Heart
Kidney
Liver
B
Cancer (markerDB)
Anova (disease association), p = 1.6e-05
0.034
0.034
0.034
0.06
MCS
n = 16
n = 105
n = 15
n = 111
n = 16
n = 110
n = 16
n = 116
Brain
Heart
Kidney
Liver
Disease association
associated
non-associated

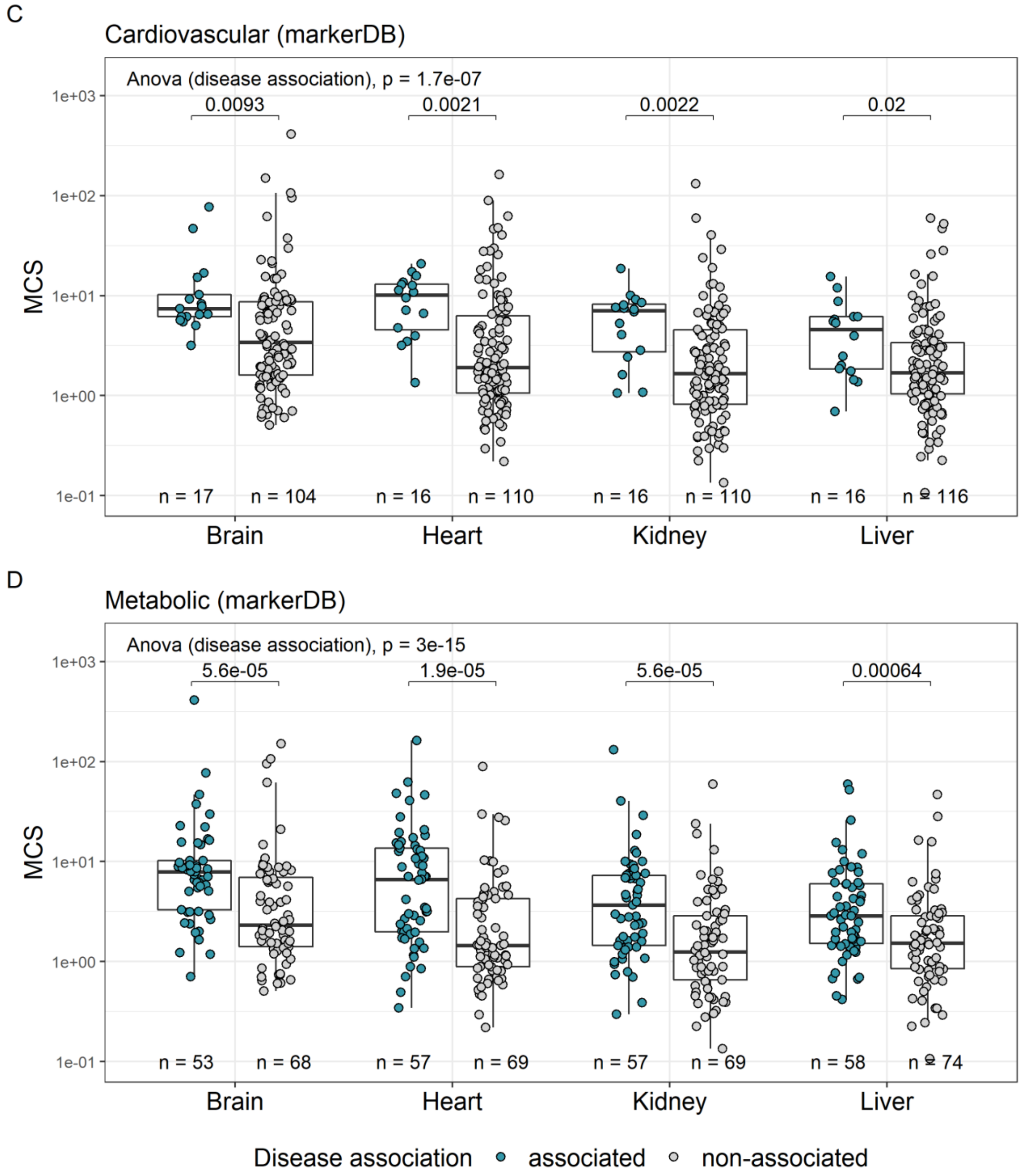

Figure S12: Metabolites associated with certain disease conditions show high MCS in all four studied organs. Broad disease conditions are as follows: **A**: Inborn errors of metabolism (IEM) associated metabolites according to our manual curation of the literature, **B**: cancer-associated metabolites according to MarkerDB, **C**: cardiovascular

disease associated metabolites according to MarkerDB and **D**: metabolic disease associated metabolites according to MarkerDB. P-values corresponding to each individual organ are shown above the boxplots and were calculated using the two-sided Wilcoxon rank sum test.

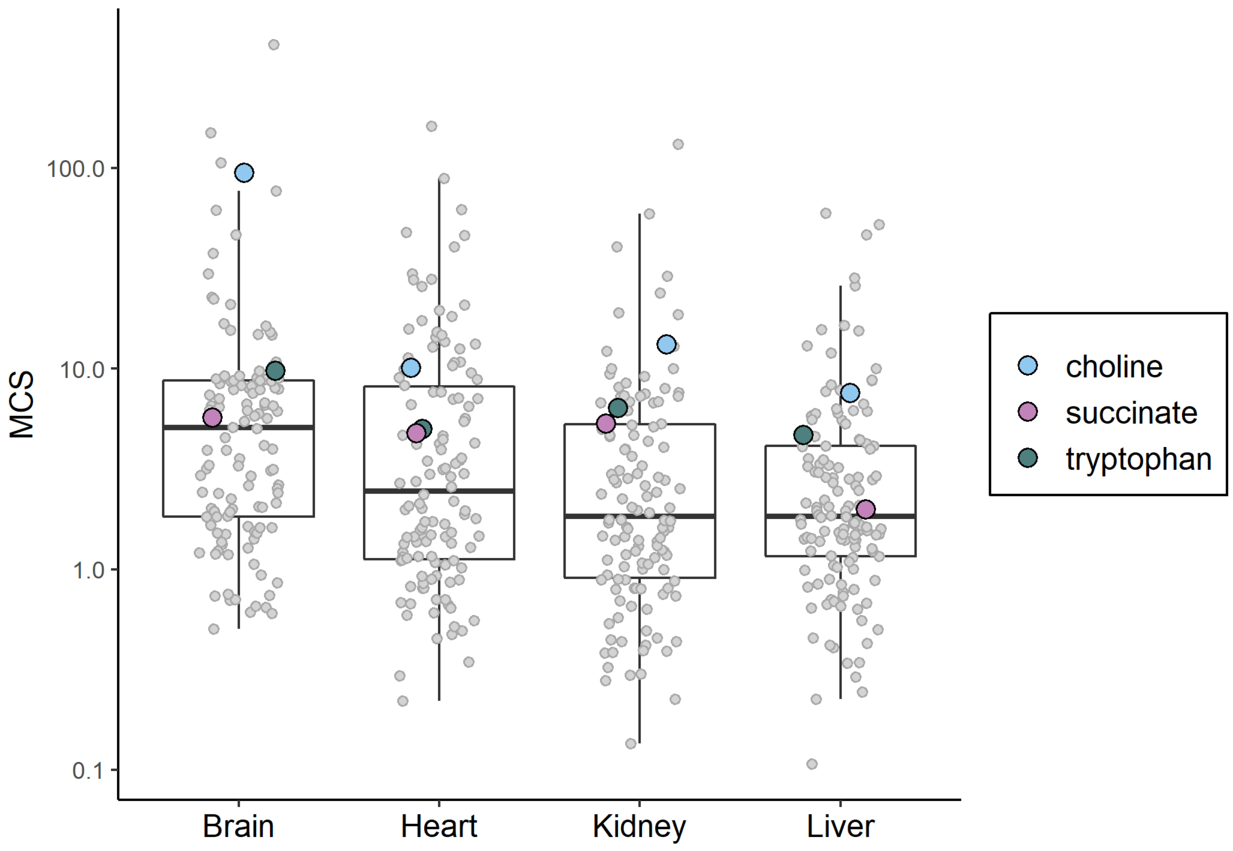

Figure S13: **Conservation scores of three metabolites associated with cancers that are not involved in IEMs.** Plot shows the conservation scores of all measured metabolites in the four organs (grey dots), with three specific metabolites marked with colors: choline and tryptophan are associated with several types of cancers, while succinate is a known oncometabolite and has a role in cardiovascular conditions as well. Importantly, none of the three highlighted metabolites are associated with metabolic disorders according to MarkerDB.

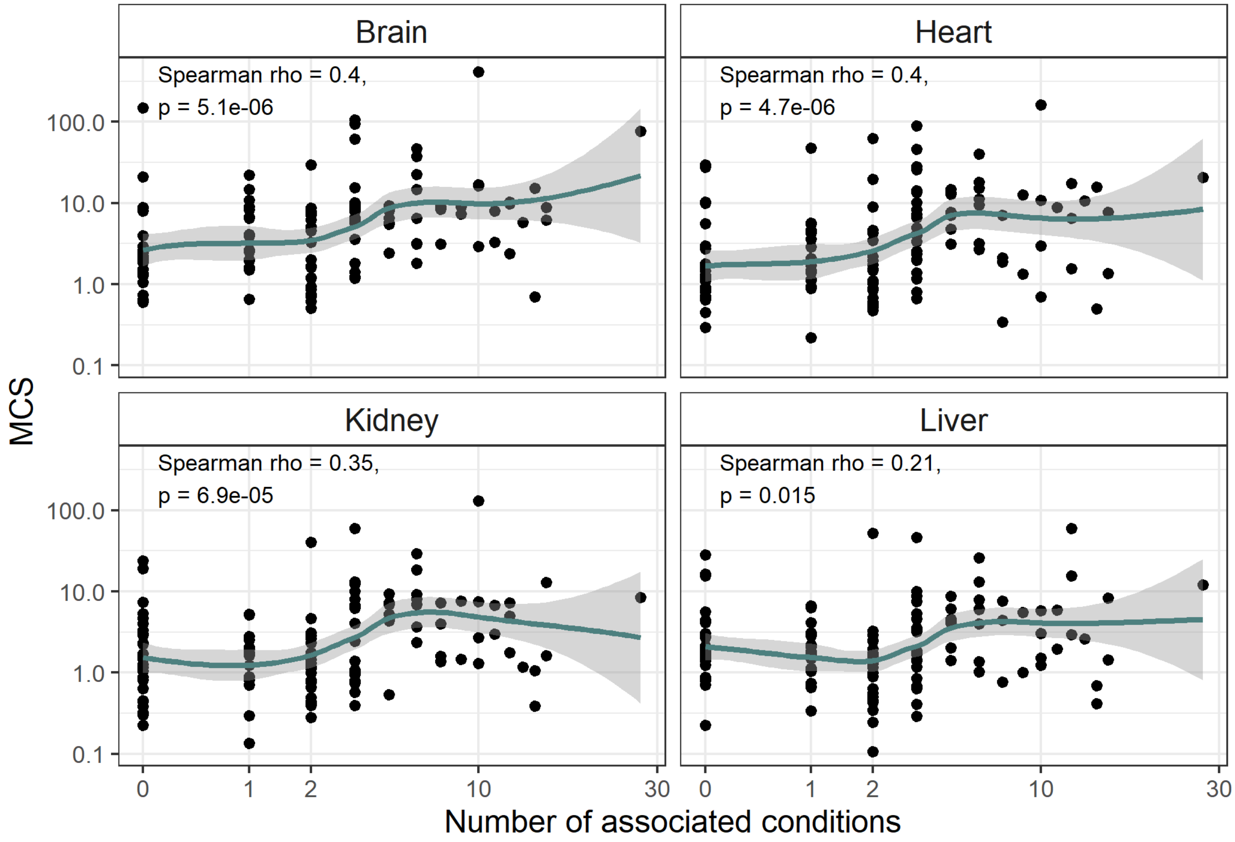

Figure S14: Metabolites associated with many diseases display high conservation scores in mammals. Scatterplots show the associations between conservation scores of metabolites and the number of specific disease conditions they are associated with, according to MarkerDB (N_{Brain}=121, N_{Heart}=126, N_{Kidney}=126, N_{Liver}=132). Correlations are significant in all four studied organs (Spearman's rho). Lines indicate smooth curve fitted using LOESS regression, with their 95% confidence intervals shaded in grey.

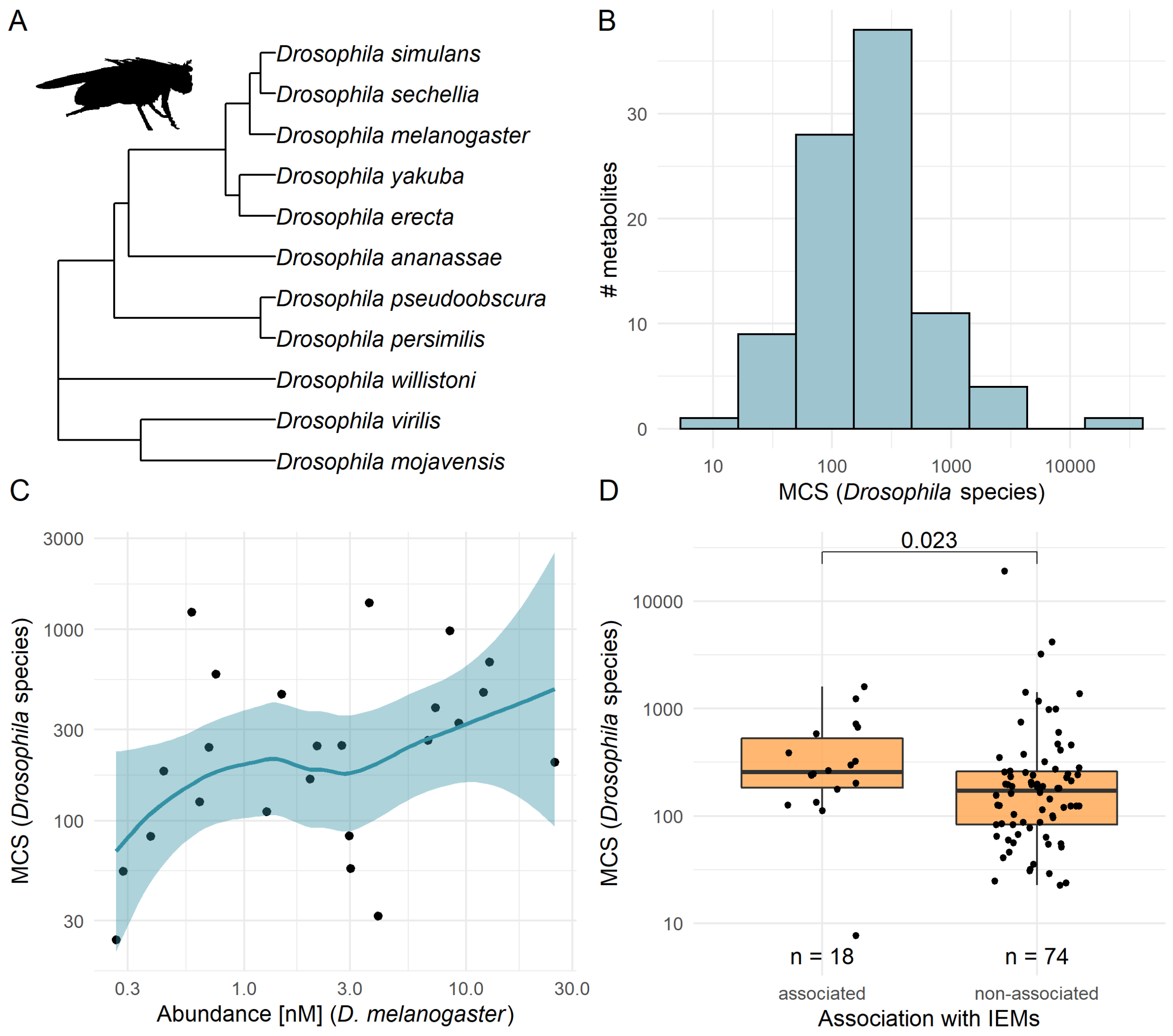

Figure S15. Metabolite concentration conservation in the Drosophila genus. A: Phylogenetic tree of 11 Drosophila species included in the analysis, according to (8). **B**: Distribution of metabolite conservation scores across 92 metabolites, calculated for the Drosophila genus. **C**: Metabolites with a high absolute concentration (abundance) in D. melanogaster tend to show high conservation score in Drosophila (Spearman's correlation rho = 0.43, p = 0.037, N=24). **D**: Metabolites associated with inborn errors of metabolism in human have an elevated conservation score in Drosophila (two-sided Wilcoxon rank sum test, p-value shown in figure).

Supplementary Tables

Table S1: The percentage of variance in metabolite conservation score explained by individual metabolite features. The table also shows the directions and p-values of the effects. “R^2 of metabolite feature” indicates the proportion of variance in MCS explained by each metabolite feature in a multiple linear regression model including both the metabolite feature and organ membership as explanatory variables to account for between-organ effects. P-value refers to significance level of the metabolite feature while accounting for organ membership. Metabolite features that have a significant effect on conservation score following false discovery rate (FDR) correction when accounting for organ membership were included in the multivariate analysis.

Feature group	Metabolite feature name	Direction	R^2 of metabolite feature	FDR-adjusted p-value
**Essentiality**	Essentiality	positive	0.078	4.57E-08
**Position in metabolic network**	In biosynthetic reaction	positive	0.028	0.0013
	In degradation reaction	negative	2.60E-04	0.75
	In energy metabolism	negative	0.0086	0.078
**Regulation**	Is activator metabolite	positive	0.054	2.39E-07
	Is inhibitor metabolite	negative	3.76E-04	0.68
	Is co-factor	negative	0.0081	0.047
**Abundance**	Abundance	positive	0.18	6.03E-15
**Hydrophobicity**	Hydrophobicity	negative	0.0048	0.13
**Solubility**	Solubility	positive	0.061	1.39E-07
**Molecular weight**	Molecular weight	negative	0.082	5.49E-10
**pKa**	pKa (acidic)	negative	0.0033	0.22
	pKa (basic)	positive	0.0083	0.060
**Network degree**	Network degree	positive	0.031	2.54E-04
**Toxicity (LD50)**	Toxicity (LD50)	negative	0.037	0.011
**Metabolite class**	Aliphatic compounds	positive	0.00015	0.78
	Amino acids	positive	0.024	0.00085
	Aromatic compounds	negative	0.014	0.0076
	Carbohydrates	negative	0.023	0.00085
	Nucleosides, nucleotides	negative	0.032	0.00021
	Organic acids	positive	0.023	0.00085
	Other metabolites	positive	0.016	0.0044
**Pathway association (KEGG)**	TCA cycle (hsa00020)	positive	0.0021	0.51
	Pentose and glucoronate interconversions (hsa00040)	positive	0.00065	0.69
	Galactose metabolism (hsa00052)	negative	0.0032	0.38

	Ascorbate and aldarate metabolism (hsa00053)	positive	0.0018	0.52
	Arginine biosynthesis (hsa00220)	positive	0.019	0.017
	Purine metabolism (hsa00230)	negative	0.002	0.51
	Pyrimidine metabolism (hsa00240)	negative	0.0042	0.31
	Alanine, aspartate, glutamate metabolism (hsa00250)	positive	0.009	0.12
	Glycine, serine, threonine metabolism (hsa00260)	positive	0.032	0.0015
	Cysteine, methionine metabolism (hsa00270)	positive	0.0013	0.58
	Valine, leucine, isoleucine biosynthesis (hsa00290)	positive	0.026	0.0036
	Lysine degradation (hsa00310)	positive	0.0068	0.21
	Arginine, proline metabolism (hsa00330)	positive	0.0099	0.11
	Histidine metabolism (hsa00340)	positive	0.00079	0.67
	Tyrosine metabolism (hsa00350)	positive	0.0058	0.23
	Phenylalanine metabolism (hsa00360)	positive	0.005	0.27
	Tryptophan metabolism (hsa00380)	negative	0.017	0.02
	Phenylalanine, tyrosine, tryptophan biosynthesis (hsa00400)	positive	0.00026	0.76
	Beta-alanine metabolism (hsa00410)	negative	0.00032	0.76
	Taurine, hypotaurine metabolism (hsa00430)	positive	0.03	0.0015
	Glutathione metabolism (hsa00480)	positive	0.00019	0.77
	Pyruvate metabolism (hsa00620)	positive	0.018	0.018
	Glyoxalate, dicarboxylate metabolism (hsa00630)	positive	0.043	0.00019
	Butanoate metabolism (hsa00650)	positive	0.0012	0.58
	Thiamine metabolism (hsa00730)	positive	0.0063	0.22
	Nicotinate and nicotinamide metabolism (hsa00760)	negative	0.00025	0.76
	Pantothenate and CoA metabolism (hsa00770)	positive	0.004	0.31

Table S2: **Estimating the upper limit of predictability of metabolite conservation score.** To estimate the upper limit of predictability of MCS, we calculated the proportion of variance in MCS explained by MCS calculated on independent clades on the phylogenetic tree, yielding an estimate of the evolutionary reproducibility of metabolite conservation. In the table, “R^2 of clade-specific MCS“ denotes the proportion of variance in MCS calculated in one clade explained by MCS calculated on the other clade using a multiple linear regression model including both clade-specific MCS and organ membership as explanatory variables to account for between-organ effects. P-value refers to significance level of the clade-specific MCS while accounting for organ membership.

**Response variable**	**Explanatory variable**	**R^2 of clade-specific MCS**	**p-value**
MCS calculated in Clade 1	MCS calculated in Clade 2	0.49	3.9E-82
MCS calculated in Clade 2	MCS calculated in Clade 1	0.48	3.9E-82

Table S3: Statistics of the most parsimonious multivariate metabolite feature model. The table contains the statistics of the most parsimonious multivariate model, a linear model comprised of the 7 metabolite features which best predict the extent of evolutionary conservation, as well as organ membership. The last two rows contain the independent effect of organ membership (R^2) and the portion of variance in MCS (R^2) jointly explained by the 7 metabolite features. The independent effect of organ membership was estimated by fitting a simpler multivariate model without organ membership and calculating the decrease in the adjusted R^2 value compared to the full multivariate model. The portion of variance explained by the metabolite features was determined by subtracting the independent effect of organ membership from the adjusted R^2 of the full model (Methods).

Residual standard error	0.46
Adjusted R^2 of full model	0.4
F- statistic	12.32
p-value	2.14e-22
Effect of organ membership (R^2)	0.091
Adjusted R^2 of metabolite feature effects	0.31

Table S4: Metabolite features included in the multivariate regression models. The table shows all the metabolite features that individually showed a significant association with conservation score (and were therefore included in the multivariate analysis). Five metabolite features and three KEGG pathways were included in the final model, following stepwise model selection. The statistical significance of these features and their independent effects in the final model are also indicated.

Metabolite feature	Included in initial multivariate model	Included in final multivariate model	Significant in final model ($p<0.05$)	Effect of predictor (R^2)
Abundance	X	X	Yes	0.12
Essentiality	X	X	Yes	0.033
Class	X	X	No	0.016
Molecular weight	X	X	Yes	0.040
Solubility	X	-	-	-
Network degree	X	-	-	-
Biosynthesis	X	-	-	-
Activator	X	-	-	-
Arginine biosynthesis (hsa00220)	X	-	-	-
Glycine, serine and threonine metabolism (hsa00260)	X	X	No	0.0062
Valine, leucine and isoleucine biosynthesis (hsa00290)	X	X	No	0.0023
Tryptophan metabolism (hsa00380)	X	-	-	-
Taurine and hypotaurine metabolism (hsa00430)	X	-	-	-
Pyruvate metabolism (hsa00620)	X	-	-	-
Glyoxylate and dicarboxylate metabolism (hsa00630)	X	X	No	0.0058

Table S5: Spearman correlation between metabolite abundance and measurement error adjusted conservation scores. The positive relationship between evolutionary conservation and metabolite abundance holds even when calculating MCS while explicitly accounting for metabolite measurement variability on the species-level (based on biological replicate measurements).

Organ	Spearman correlation rho	Spearman correlation p-value
Brain	0.34	0.0033.
Heart	0.43	0.00032.
Kidney	0.53	2.023e-05.
Liver	0.36	0.0028.

Table S6: Metabolites showing organ-specific conservation. The table lists all metabolites that show the top 10% highest organ-specific deviation in conservation score in at least one organ, along with the specific organ(s) in which they are strongly conserved and their organ-specific effect size scores in brain, heart, kidney and liver. A larger deviation score indicates that a metabolite is more conserved in the given organ than in the others.

Metabolite	Most conserved organ	Organ-specific deviation in conservation score			
		Brain	Heart	Kidney	Liver
hypoxanthine	Brain, Heart, Kidney	2.85	1.91	2.13	-6.89
citrate	Brain	2.71	-2.70	0.58	-0.59
trimethylamine-N-oxide	Brain	2.60	-0.31	-1.31	-0.98
choline	Brain	2.30	-1.14	-0.02	-1.13
DHAP/glyceraldehyde 3P	Brain	2.29	0.03	-0.82	-1.50
xanthine	Brain	2.23	-2.95	0.26	0.45
glutamate	Brain	2.21	0.35	0.57	-3.13
creatine	Brain, Heart	2.13	2.66	-1.71	-3.09
aconitate	Brain	1.93	-1.41	-0.97	0.45
GABA	Brain	1.91	-0.90	-0.12	-0.89
arginine	Brain, Heart	1.80	2.74	-1.83	-2.71
taurine	Heart	-1.12	2.96	1.02	-2.86
inosine	Heart	0.61	2.95	-0.68	-2.89
pyroglutamic acid	Heart	-1.02	2.43	0.30	-1.71
carnitine	Heart	-0.63	2.18	-0.62	-0.93
pyruvate	Heart	-0.09	1.96	-1.66	-0.20
putrescine	Heart	1.41	1.92	-2.57	-0.76
phosphocholine	Heart	0.20	1.60	-1.46	-0.34
adipate	Heart	0.77	1.45	-1.56	-0.66
betaine	Kidney	-1.91	-1.04	2.99	-0.04
xanthosine	Kidney	0.18	-2.03	2.23	-0.37
glucuronate	Kidney	-1.76	-1.15	2.23	0.68
hexose diphosphate	Kidney	-1.36	-0.64	1.74	0.27
adenine	Kidney	-1.21	-0.80	1.50	0.51
inositol	Kidney	0.76	-1.32	1.40	-0.84
cystathionine	Kidney	-1.01	-0.79	1.25	0.56
glycine	Kidney	-1.03	-0.44	1.18	0.28
aspartate	Kidney	-0.96	0.54	1.09	-0.66
citrulline	Kidney	-1.65	-0.28	1.07	0.86
fructose/glucose/galactose	Liver	-2.64	-2.59	0.26	4.98

uracil	Liver	-1.42	-0.86	-0.47	2.75
3-phosphoglycerate	Liver	-1.33	0.16	-1.16	2.33
hexose monophosphate	Liver	-1.46	-0.28	-0.40	2.13
phosphoenolpyruvate	Liver	-0.14	-0.05	-1.64	1.82
kynurenine	Liver	-0.96	-0.53	-0.24	1.73
NADP	Liver	-1.33	-0.92	0.59	1.65
4-pyridoxate	Liver	-1.76	-0.30	0.47	1.59
lactose	Liver	0.43	-0.21	-1.70	1.48
dUMP	Liver	-1.37	-0.01	-0.05	1.44
GDP	Liver	-0.11	-2.02	0.71	1.41

Table S7: Independent effects of metabolite abundance and reaction essentiality on metabolite conservation score in the evolutionary simulations.

Here we tested whether two metabolite properties, metabolite abundance and involvement in essential reaction, are independently associated with metabolite conservation scores in the presence of stabilizing selection in the erythrocyte metabolic model. For 32 metabolites, metabolite abundance and involvement in essential reaction were calculated from biochemical modelling of the wild-type erythrocyte network, while metabolite conservation scores were calculated from evolutionary simulations (see Methods). We used multivariate linear modelling to predict metabolite conservation score based on metabolite abundance and involvement in essential reaction. Note that metabolite conservation scores and metabolite abundances were log-transformed, while involvement in essential reaction was a binary variable. A linear model was fitted using the R function lm() and explained well variation in conservation scores across metabolites (adjusted R-squared: 0.65, p-value = 7.70e-08). Both involvement in essential reaction and metabolite abundance showed a highly significant effect in the multivariate model, indicating that these properties independently determine metabolite conservation score.

Explanatory variable	Estimate	Standard error	t-value	P-value	
(Intercept)	10.12	0.49	21.57	< 2e-16	***
In essential reaction	1.85	0.42	4.35	1.55e-04	***
Metabolite abundance	0.81	0.15	5.23	1.35e-05	***

Table S8: Metabolites associated with inborn errors of metabolism. Only the metabolites present in our evolutionary dataset are shown.

Disease name	OMIM ID	Orphanet ORPHAcode	Causative gene name	Gene NCBI ID	Associated metabolite name	Associated metabolite HMDB ID
Propionic Acidemia	606054	ORPHA:35	PCCA; PCCB	5095; 5096	propionate	HMDB0000237
Methylmalonic Acidemia (methylmalonyl-CoA mutase)	251000	ORPHA:27; ORPHA:79312; ORPHA:289916	MMUT	4594	propionate	HMDB0000237
Methylmalonic Acidemia (Cobalamin disorders)	277410; 251100; 251110; 251120	ORPHA:26; ORPHA:28; ORPHA:622; ORPHA:79283; ORPHA:79310; ORPHA:79311; ORPHA:308380; ORPHA:308442; ORPHA:308425	MMAA; MMAB ;MMADHC; MCEE	166785; 326625; 27249; 84693	propionate	HMDB0000237
Isovaleric Acidemia	243500	ORPHA:33	IVD	3712		
3-Methylcrotonyl-CoA Carboxylase Deficiency	210200; 210210	ORPHA:6	MCCC1; MCCC2	56922; 64087		
3-Hydroxy-3-Methyglutaric Aciduria	246450	ORPHA:20	HMGCL	3155		
Holocarboxylase Synthase Deficiency	253270	ORPHA:79242	HLCS	3141	lactate	HMDB0000190
beta-Ketothiolase Deficiency	203750	ORPHA:134	ACAT1	38		
Glutaric acidemia	231670	ORPHA:25	GCDH	2639		
Carnitine Uptake Defect/Carnitine Transport Defect	212140	ORPHA:158	SLC22A5	6584	carnitine	HMDB0000062
Medium-chain Acyl-CoA Dehydrogenase Deficiency	201450	ORPHA:42	ACADM	34		
Very Long-chain Acyl-CoA Dehydrogenase Deficiency	201475	ORPHA:26793	ACADVL	37		
Long-chain L-3 Hydroxyacyl-CoA Dehydrogenase Deficiency	609016	ORPHA:5	HADHA	3030	suberate	HMDB0000893
					adipate	HMDB0000448
Trifunctional Protein Deficiency	609015	ORPHA:746	HADHA; HADHB	3030; 3032	suberate	HMDB0000893
					adipate	HMDB0000448
Argininosuccinic Aciduria	207900	ORPHA:23	ASL	435	alanine	HMDB0000161
					citrulline	HMDB0000904
					glutamine	HMDB0000641
					glycine	HMDB0000123
Citrullinemia, Type I	215700	ORPHA:247525	ASS1	445	citrulline	HMDB0000904
					orotate	HMDB0000226
Maple Syrup Urine Disease	248600	ORPHA:511; ORPHA:268145; ORPHA:268162;	BCKDHA; BCKDHB; DBT	593; 594; 1629	fumarate/maleate /alpha-ketoisovalerate	HMDB0000019
					isoleucine	HMDB0000172

		ORPHA:268173; ORPHA:268184			leucine	HMDB0000687
					valine	HMDB0000883
Homocystinuria	236200; 236250; 236270; 250940	ORPHA:394; ORPHA:395; ORPHA:622; ORPHA:2169; ORPHA:2170	CBS; MTHFR; MTR; MTRR	875; 4524; 4548; 4552	methionine	HMDB0000696
Classic Phenylketonuria	261600	ORPHA:716; ORPHA:2209	PAH	5053	phenylalanine	HMDB0000159
Tyrosinemia, Type I	276700	ORPHA:882	FAH	2184	tyrosine	HMDB0000158
Biotinidase Deficiency	253260	ORPHA:79241	BTD	686		
Classic Galactosemia	230400	ORPHA:352; ORPHA:79239	GALT	2592	fructose/glucose/ galactose	HMDB0000143
Glycogen Storage Disease Type II (Pompe disease)	232300	ORPHA:365	GAA	2548		
Mucopolysaccharidosis Type 1	607014; 607015; 607016	ORPHA:579; ORPHA:93473; ORPHA:93474; ORPHA:93476	IDUA	3425		

Table S9: Metabolites associated with inborn errors of metabolism show elevated conservation scores independent of amino acid and metabolic pathway overrepresentations and the effect of metabolite abundance.

We tested whether IEM-associated metabolites have higher metabolite conservation scores than the rest of metabolites under four scenarios: (i) for the entire dataset, (ii) when removing all amino acids from the dataset, (iii) when removing metabolites involved in one of the pathways containing three or more IEM-associated metabolites, namely *Arginine biosynthesis* (hsa00220) and *Valine, leucine and isoleucine biosynthesis* (hsa00290), and (iv) when controlling for the effect of metabolite abundance. In the first three cases, we used ANOVA models accounting for both organ membership and IEM-association, while the effect of metabolite abundance was controlled for by building a linear model with both abundance and IEM-association as explanatory variables. IEM-associated metabolites were significantly more conserved in all cases, and both abundance and IEM-association had significant independent effects on metabolite conservation score.

**Test**	**Explanatory variable**	**Effect of IEM-association**		
		**F-value**	**p-value**	
All IEM associations	IEM-association	55.82	3.6e-13	***
No amino acids	IEM-association	25.29	8e-07	***
No *hsa00220* metabolites	IEM-association	54.4	7.07e-13	***
No *hsa00290* metabolites	IEM-association	44.47	7.03e-11	***
Corrected for abundance	IEM association	37.6	2.7e-09	***
	Abundance	20.79	7.4e-06	***

Table S10: Testing the associations between MarkerDB disorder categories and metabolite conservation in a multivariate model. We used a multivariate linear regression model to identify broad disorder categories whose biomarkers show an elevated conservation score compared to the rest of metabolites. Note that only disorder categories containing at least ten metabolites from our dataset and showing a significant association with conservation score (single-variable two-sided Wilcoxon rank sum test $p<0.05$) in at least three organs were included in the multivariate linear regression model.

**Disease category**	**# of metabolites**	**# of significant tissues**	**Included in the multivariate model**	**Significant relationship between MCS and disorder category in the multivariate model ($p<0.05$)**
Cancer	16	3	X	Yes
Cardiovascular system disorder	17	3	X	Yes
Digestive system disorder	33	2	-	-
Endocrine system disorder	16	0	-	-
Germline disorder	71	3	X	No
Hematological or lymphatic system disorder	16	1	-	-
Immune system disorder	38	1	-	-
Mental or behavioral disorder	56	2	-	-
Metabolic disorder	60	4	X	Yes
Nervous system disorder	74	0	-	-
Urinary system disorder	32	1	-	-

Table S11: Metabolites associated with many diseases are more conserved, even when accounting for metabolite abundance and essentiality. Statistics of two linear models that assess the relationship between the conservation score of metabolites and the number of human diseases they are associated with while accounting for (A) metabolite abundance and (B) metabolite essentiality. Metabolites associated with a larger number of human diseases are significantly more conserved even after controlling for the effects of metabolite abundance and essentiality. Both linear models used also included organ membership as an explanatory variable, so that the effects of disease participation and metabolite features could be assessed for all organs in a single model.

**Test**	**Variable**	**F-value**	**P-value**	
A: Controlling for abundance	Number of associated disorders	21.93	4.5e-06	***
	Metabolite abundance	49.52	1.8e-11	***
B: Controlling for essentiality	Number of associated disorders	23.46	2.02e-06	***
	Involvement in essential reactions	18.42	2.4e-05	***

Table S12: List of simulation parameters for each *in silico* evolution experiment.

Parameter name	Symbol	Genetic drift simulations	Stabilizing selection simulations
Selection threshold	ω	$+\infty$	1×10^{-4}
Mutation size	σ_{mut}	1×10^{-2}	
Number of iterations	T	$10{,}000$	
Repetitions	-	10	

Appendix S1: Evaluation of two additional kinetic models

To further test whether stabilizing selection on key metabolic fluxes explains patterns of metabolite conservation (see main text), we performed the same analyses as for the erythrocyte metabolism model (7) for two other publicly available kinetic models: another model of human erythrocyte metabolism (9) (56 variables metabolites, 53 reactions, 234 kinetic parameters) and a model of human hepatic glucose metabolism (10) (24 variable metabolites, 36 reactions, 200 kinetic parameters). The mutation size was $\sigma_{mut} = 1 \times 10^{-2}$ for all the simulations and fitness coupling analyses; the selection threshold was $\omega = 1 \times 10^{-5}$ for the erythrocyte model and $\omega = 1 \times 10^{-2}$ for the hepatocyte model. We ran the evolutionary simulations for $T = 10{,}000$ iterations (10 repetitions per experiment). Five low-varying metabolites (glutathione, NAD, NADPH, phosphate, and pyruvate) have been removed in the erythrocyte model, based on the filtering approach described in Methods. One low-varying metabolite (mitochondrial GTP) has been removed in the hepatocyte model. For the erythrocyte model, the key metabolic fluxes were ATP utilization (*vatpase*), glutathione (GSH) oxidation (*vox*) and the formation of 2,3-bisphosphoglycerate (*vbpgsp7*). For the hepatocyte model, the key metabolic fluxes were the reactions associated with blood glucose regulation: glycogenesis (fluxes *GS* and *GP*), the production of oxaloacetate from pyruvate, a precursor of gluconeogenesis (*PC*) and the production of Acetyl-CoA, a precursor of triglycerides (*PDH*).

For each kinetic model, we also computed the fitness coupling of metabolites for 10,000 random combinations of key fluxes, and we computed stabilizing selection simulations for 100 random combinations of key fluxes (from 1-uplets up to 3-uplets for the erythrocyte model; from 1-uplets to 4-uplets for the hepatocyte model). We also computed a flux inhibition analysis for each model to determine essential reactions and associated metabolites (see Methods). Fluxes were constrained to 1% of their wild-type levels for the erythrocyte model (numerical instability for flux values close to zero did not allow us to use lower values). Fluxes were constrained to 0.001% for the hepatocyte model.

We also found that metabolite abundances positively correlate with their conservation scores under stabilizing selection, but not under genetic drift (Figs App. 1A-B, App. 2A-

B), and that abundant metabolites are significantly more coupled to fitness (Figs App. 1C, App. 2C). Essential metabolites have significantly higher conservation scores compared to non-essential metabolites under stabilizing selection, but not under genetic drift (Figs. App. 1D-E, App. 2D-E). Furthermore, for both models, a linear regression model shows that both metabolite abundance and metabolite essentiality are independent predictors of *in silico* metabolite conservation scores under stabilizing selection (Tables App. 1 and App. 2). Similarily to the first erythrocyte model[1], the key metabolic fluxes generally assumed to be important for the fitness are significantly more coupled to abundant metabolites than 99% of 10,000 random combinations of key fluxes for both additional models (Figs App. 1F, App. 2F). Moreover, when evolution was simulated under stabilizing selection for 100 models with randomly defined key fluxes, we again found that metabolite conservation score correlates more strongly with abundance in those models in which abundant metabolites are strongly coupled to fitness (Figs App. 1G, App. 2G).

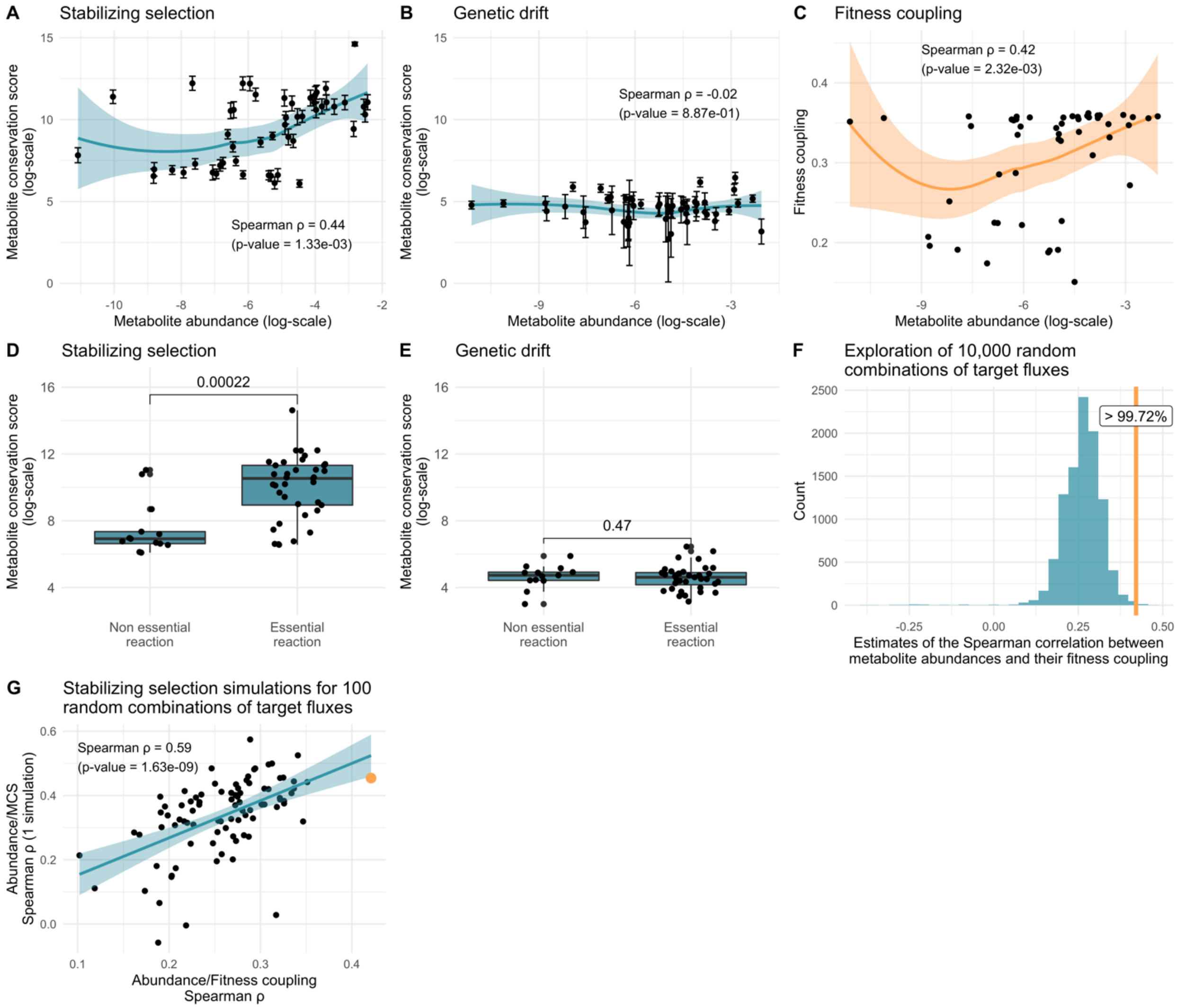

Figure App. 1: Functional constraints in the human erythrocyte metabolism model (9). A-B: Metabolites with a higher abundance in the wild-type model show higher conservation scores in the presence of stabilizing selection (panel A, Spearman $\rho = 0.44$, $p = 1.33 \times 10^{-3}$, $N = 51$), but not in the absence of selection (panel B, Spearman $\rho = -0.02$, $p = 0.887$, $N = 51$). Each dot and error bar represents the mean and standard deviation of metabolite conservation scores of a particular metabolite based on 10 simulations. Blue lines represent LOESS regressions, with their 95% confidence interval in shaded blue. C: Wild-type abundances of metabolites correlate with their extent of fitness coupling (Spearman $\rho = 0.49$, $p = 3.14 \times 10^{-3}$). The orange line represents a LOESS regression, with its 95% confidence interval in shaded orange. D-E: Metabolites involved in essential reactions show higher conservation scores than those involved in non-essential reactions

in the presence of stabilizing selection (panel D, two-sided Wilcoxon test $p = 2.22 \times 10^{-4}$), but not in the absence of selection (panel E, two-sided Wilcoxon test $p = 0.393$). Boxplots show the median, first and third quartiles, with the whiskers showing the values within a 1.5 inter-quartile range distance from the 1st and 3rd quartiles. Each dot represents the mean conservation score of a particular metabolite based on 10 simulations. Involved metabolites are reactants and products of the reaction (see Methods) F: The fitness coupling of metabolite abundances has been computed for 10,000 random combinations of key fluxes (uniformly distributed from 1-uplets to 3-uplets). Then, the Spearman correlation is computed between metabolite abundance and fitness coupling across metabolites. The distribution of the 10,000 Spearman estimates is shown. The orange line represents the Spearman estimate of the default key fluxes (vatpase, vox, vbpgsp7), which is higher than 99.72% of the 10,000 random combinations. G: Stabilizing selection simulations have been run for 100 random combinations of key fluxes. At the end of each simulation, an *in silico* conservation score was calculated for each metabolite. Then, for each simulation, we plotted the Spearman correlation estimate between metabolite abundance and conservation score across metabolites against the Spearman estimate of the correlation between metabolite abundance and fitness coupling across metabolites. The two Spearman estimates are significantly positively correlated (Spearman $\rho = 0.59$, $p = 1.63 \times 10^{-9}$). The bold orange dot represents the Spearman estimates for the default key fluxes (vatpase, vox, vbpgsp7).

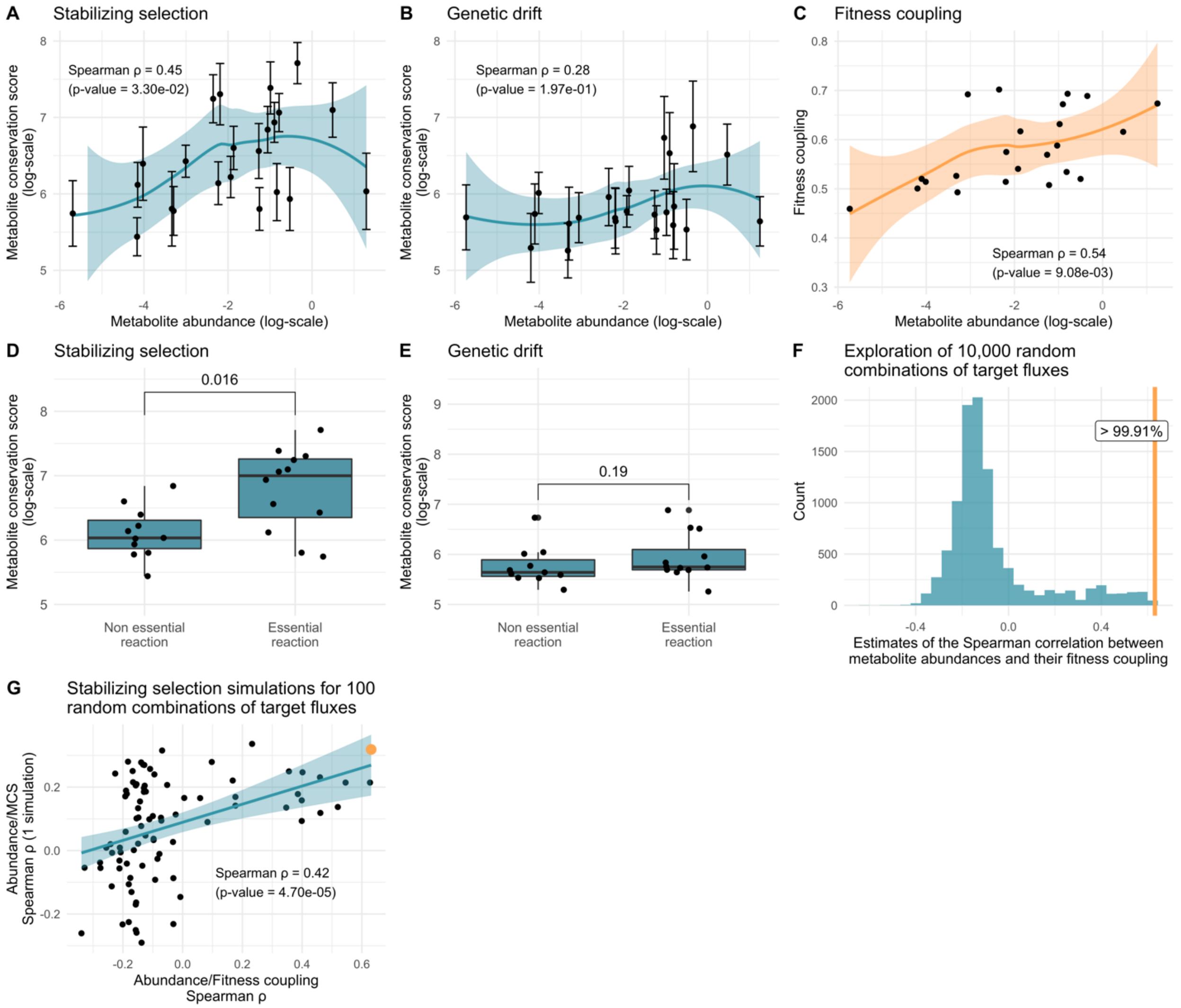

Figure App. 2: Functional constraints in the human hepatic glucose metabolism model (10). A-B: Metabolites with a higher abundance in the wild-type model show higher conservation scores in the presence of stabilizing selection (panel A, Spearman $\rho = 0.45$, p $= 3.30 \times 10^{-2}$, $N = 23$), but not in the absence of selection (panel B, Spearman $\rho = 0.28$, p $= 0.197$, $N = 23$). Each dot and error bar represents the mean and standard deviation of evolutionary rate of a particular metabolite based on 10 simulations. Blue lines represent LOESS regressions, with their 95% confidence interval in shaded blue. C: Wild-type abundances of metabolites correlate with their extent of fitness coupling (Spearman $\rho = 0.52$, p $= 1.08 \times 10^{-2}$). The orange line represents a LOESS regression, with its 95% confidence interval in shaded orange. D-E: Metabolites involved in essential reactions show higher conservation scores than those involved in non-essential reactions in the presence of stabilizing selection (panel D, two-sided Wilcoxon test p $= 0.016$), but

not in the absence of selection (panel E, two-sided Wilcoxon test $p = 0.190$). Boxplots show the median, first and third quartiles, with the whiskers showing the values within a 1.5 inter-quartile range distance from the 1st and 3rd quartiles. Each dot represents the mean conservation score of a particular metabolite based on 10 simulations. Involved metabolites are reactants and products of the reaction (see Methods). F: The fitness coupling of metabolite abundances has been computed for 10,000 random combinations of key fluxes (uniformly distributed from 1-uplets to 4-uplets). Then, the Spearman correlation is computed between metabolite abundances and their fitness coupling. The distribution of the 10,000 Spearman estimates is shown. The orange line represents the Spearman estimate of the default key fluxes (*GS, GP, PC, PDH*), which is higher than 99.91% of the 10,000 random combinations. G. Stabilizing selection simulations have been run for 100 random combinations of key fluxes. At the end of each simulation, an *in silico* conservation score was calculated for each metabolite. Then, for each simulation, we plotted the Spearman correlation estimate between metabolite abundance and conservation score across metabolites against the Spearman estimate of the correlation between metabolite abundance and fitness coupling across metabolites. The two Spearman estimates are significantly positively correlated (Spearman $\rho = 0.42$, $p = 4.53 \times 10^{-5}$). The bold orange dot represents the Spearman estimates for the default key fluxes (*GS, GP, PC, PDH*).

Table App. 1: For the erythrocyte model (9), a linear regression model shows that metabolite abundance and metabolite essentiality are predictors of *in silico* metabolite conservation score under stabilizing selection. The function lm (R 4.0.0) is used with no interaction terms on log-transformed metabolite conservation scores and metabolite abundances (metabolite essentiality being a categorical variable). Adjusted R-squared: 0.3701, p-value < 7.203e-06 (p-value stars: "*": p < 0.05, "**": p < 0.01, "***": p < 0.001).

**Explanatory variable**	**Estimate**	**Standard error**	**t value**	**P-value**	
(Intercept)	9.9651	0.9171	10.866	2.05e-14	***
Metabolite essentiality	2.1493	0.5579	3.852	0.000354	***
Metabolite abundance	0.3939	0.1272	3.098	0.003289	**

Table App. 2: For the hepatocyte model (10), a linear regression model shows that metabolite abundance and metabolite essentiality are predictors of *in silico* metabolite conservation score under stabilizing selection. The function lm (R 4.0.0) is used with no interaction terms on log-transformed metabolite conservation scores and metabolite abundances (metabolite essentiality being a categorical variable). Adjusted R-squared: 0.4877, p-value = 4.81e-4 (p-value stars: "*": p < 0.05, "**": p < 0.01, "***": p < 0.001).

**Explanatory variable**	**Estimate**	**Standard error**	**t value**	**P-value**	
(Intercept)	6.44448	0.17188	37.495	< 2e-16	***
Metabolite essentiality	0.71701	0.18879	3.798	0.00113	**
Metabolite abundance	0.18502	0.05809	3.185	0.00465	**

References

1. S. Ma, *et al.*, Organization of the Mammalian Metabolome according to Organ Function, Lineage Specialization, and Longevity. *Cell Metab* **22**, 332–343 (2015).

2. M. Sugimoto, *et al.*, MMMDB: Mouse Multiple Tissue Metabolome Database. *Nucleic Acids Research* **40**, D809–D814 (2012).

3. S. Ma, *et al.*, Cell culture-based profiling across mammals reveals DNA repair and metabolism as determinants of species longevity. *eLife* **5**, e19130 (2016).

4. J. O. Park, *et al.*, Metabolite concentrations, fluxes and free energies imply efficient enzyme usage. *Nature Chemical Biology* **12**, 482–489 (2016).

5. M. Hucka, *et al.*, The systems biology markup language (SBML): a medium for representation and exchange of biochemical network models. *Bioinformatics* **19**, 524–531 (2003).

6. S. Hoops, *et al.*, COPASI—a COmplex PAthway SImulator. *Bioinformatics* **22**, 3067–3074 (2006).

7. H.-G. Holzhütter, The principle of flux minimization and its application to estimate stationary fluxes in metabolic networks. *European Journal of Biochemistry* **271**, 2905–2922 (2004).

8. B. R. Harrison, J. M. Hoffman, A. Samuelson, D. Raftery, D. E. L. Promislow, Modular Evolution of the Drosophila Metabolome. *Molecular Biology and Evolution* (2021) https:/doi.org/10.1093/molbev/msab307 (October 25, 2021).

9. P. J. MULQUINEY, W. A. BUBB, P. W. KUCHEL, Model of 2,3-bisphosphoglycerate metabolism in the human erythrocyte based on detailed enzyme kinetic equations1: in vivo kinetic characterization of 2,3-bisphosphoglycerate synthase/phosphatase using 13C and 31P NMR. *Biochemical Journal* **342**, 567–580 (1999).

10. M. König, S. Bulik, H.-G. Holzhütter, Quantifying the Contribution of the Liver to Glucose Homeostasis: A Detailed Kinetic Model of Human Hepatic Glucose Metabolism. *PLOS Computational Biology* **8**, e1002577 (2012).
